# A Comprehensive Evaluation of IoT Cloud Platforms: A Feature-Driven Review with a Decision-Making Tool

**DOI:** 10.3390/s25165124

**Published:** 2025-08-18

**Authors:** Ioannis Chrysovalantis Panagou, Stylianos Katsoulis, Evangelos Nannos, Fotios Zantalis, Grigorios Koulouras

**Affiliations:** TelSiP Research Laboratory, Department of Electrical and Electronic Engineering, School of Engineering, University of West Attica, Ancient Olive Grove Campus, 250 Thivon Str., GR-12241 Athens, Greece; ipanagou@uniwa.gr (I.C.P.); skatsoulis@uniwa.gr (S.K.); enannos@uniwa.gr (E.N.); fzantalis@uniwa.gr (F.Z.)

**Keywords:** IoT, Cloud Platforms, feature evaluation, decision-making tool, comparative analysis, security, scalability, interoperability, data analytics, edge computing

## Abstract

The rapid proliferation of Internet of Things (IoT) devices has led to a growing ecosystem of Cloud Platforms designed to manage, process, and analyze IoT data. Selecting the optimal IoT Cloud Platform is a critical decision for businesses and developers, yet it presents a significant challenge due to the diverse range of features, pricing models, and architectural nuances. This manuscript presents a comprehensive, feature-driven review of twelve prominent IoT Cloud Platforms, including AWS IoT Core, IoT on Google Cloud Platform, and Microsoft Azure IoT Hub among others. We meticulously analyze each platform across nine key features: Security, Scalability and Performance, Interoperability, Data Analytics and AI/ML Integration, Edge Computing Support, Pricing Models and Cost-effectiveness, Developer Tools and SDK Support, Compliance and Standards, and Over-The-Air (OTA) Update Capabilities. For each feature, platforms are quantitatively scored (1–10) based on an in-depth assessment of their capabilities and offerings at the time of research. Recognizing the dynamic nature of this domain, we present our findings in a two-dimensional table to provide a clear comparative overview. Furthermore, to empower users in their decision-making process, we introduce a novel, web-based tool for evaluating IoT Cloud Platforms, called the “IoT Cloud Platforms Selector”. This interactive tool allows users to assign personalized weights to each feature, dynamically calculating and displaying weighted scores for each platform, thereby facilitating a tailored selection process. This research provides a valuable resource for researchers, practitioners, and organizations seeking to navigate the complex landscape of IoT Cloud Platforms.

## 1. Introduction

The Internet of Things (IoT) is rapidly transforming industries such as manufacturing, healthcare, and transportation, with an estimated 70 billion connected devices expected by 2027–2029 [1]. This rapid expansion of IoT underscores the critical role of IoT Cloud Platforms in enabling scalable, secure, and intelligent connected solutions [2,3]. These platforms are indispensable for device connectivity, data management, and the extraction of valuable insights from vast IoT datasets. However, the sheer diversity and complexity of the available platforms make strategic selection a significant challenge for developers and organizations. Without a clear comparative framework, identifying the optimal platform tailored to specific project demands remains a complex endeavor.

To address this challenge, this manuscript presents a systematic and comprehensive review of twelve leading IoT Cloud Platforms. Our selection includes major proprietary services like AWS IoT Core and Microsoft Azure IoT Hub, as well as a leading open-source alternative like ThingsBoard, to provide a balanced overview of the landscape. The specific criteria for choosing these platforms are detailed in Section 3.1 to ensure a balanced and representative analysis. Our evaluation spans across nine critical features: Security, Scalability and Performance, Interoperability, Data Analytics and AI/ML Integration, Edge Computing Support, Pricing Models and Cost-effectiveness, Developer Tools and SDK Support, Compliance and Standards, and OTA Update Capabilities. Furthermore, to bridge the gap between a static analysis and individual user needs, we developed a novel web-based tool for evaluating IoT Cloud Platforms, called the “IoT Cloud Platforms Selector”. This interactive tool empowers users to personalize their evaluation by assigning weighted priorities to each feature, thereby generating tailored recommendations in real time.

The main contributions of this paper can be summarized as follows:

- A systematic and comprehensive feature-driven evaluation of twelve leading IoT Cloud Platforms across nine critical criteria. This analysis provides a structured, expert-derived snapshot of the current platform landscape.
- The design and implementation of a novel, interactive web-based tool, the “IoT Cloud Platforms Selector”. This tool translates our static analysis into a dynamic, personalized decision-support system, addressing a significant gap by enabling users to find the optimal platform based on their specific, weighted requirements.

By providing a unified framework that combines a rigorous academic review and a practical decision-support utility, this research aims to serve as an invaluable resource for researchers, developers, and companies embarking on their IoT projects.

The remainder of this manuscript is structured as follows. Section 2, “Background and Related Work”, provides essential background on IoT Cloud Platforms and reviews the existing literature, highlighting the current research gaps. Section 3, entitled “Methodology”, details the systematic approach used for platform selection, feature identification, and the scoring mechanism. Section 4 briefly introduces each of the twelve evaluated IoT Cloud Platforms. Section 5 presents the core feature-driven evaluation and comparative analysis, culminating in the comprehensive score table. Section 6 describes the design, implementation and utility of the web-based tool “IoT Cloud Platforms Selector”. A detailed discussion of key findings, limitations, and avenues for future work is provided in Section 7. Finally, Section 8 concludes the paper by summarizing its main contributions.

## 2. Background and Related Work

The ubiquitous growth of connected devices has heralded the era of the Internet of Things, fundamentally transforming various sectors, from industrial automation and smart cities to healthcare and consumer electronics [4,5]. At the architectural heart of most modern IoT deployments lie IoT Cloud Platforms, which serve as the essential middleware layer connecting diverse edge devices to advanced data processing, analytics, and application services. These platforms are responsible for critical functionalities that include device connectivity and management, data acquisition and storage, real-time data processing, analytics, machine learning integration and providing interfaces for application development and user interaction [6]. The exponential increase in IoT data volumes and the need for scalable, secure, and resilient infrastructure have cemented the cloud’s role as an indispensable component of the IoT ecosystem, as emphasized by Sharma and Obaidat [7] and Hamdan et al. [8]. The integration of Wireless Sensor Networks (WSNs) with cloud infrastructure has become a prominent paradigm, augmenting WSN capabilities and leveraging the cloud’s resources. Haseeb-Ur-Rehman et al. [9] provide a comprehensive analysis of sensor cloud frameworks, outlining their architectures, network dynamics, and research challenges in integrating WSNs with cloud environments.

The evolution of IoT Cloud Platforms has been rapid, moving beyond initial Machine-to-Machine (M2M) communication frameworks to sophisticated, multi-service offerings. Early solutions often focused on specific vertical applications or basic data acquisition. However, the demands of complex and heterogeneous IoT environments have spurred the development of comprehensive platforms that integrate a wide array of services, from identity management and data stream processing to advanced analytics and edge computing capabilities. This evolution has led to a highly competitive market, characterized by significant investments from major cloud providers and specialized IoT platform vendors alike.

The increasing number and diversity of IoT Cloud Platforms pose a significant challenge to developers, businesses, and researchers in selecting the most appropriate solution for their specific use cases. Key considerations often involve performance requirements, security postures, integration complexities, developer friendliness, long-term scalability, and crucially cost-effectiveness [10]. Without a systematic evaluation framework, the selection process can be subjective, time-consuming, and potentially lead to vendor lock-in or suboptimal system architectures.

Several research efforts have attempted to provide comparative analyses or reviews of IoT Cloud Platforms [11,12,13,14,15,16,17,18,19,20,21,22,23,24]. For instance, Ref. [12] provided an early overview of IoT enabling technologies, mentioning the role of cloud computing, but without a dedicated platform comparison. More focused studies, such as [13], offered qualitative comparisons of a limited number of platforms based on general architectural components. Similarly, ref. [14] discussed the benefits and challenges of IoT cloud integration from a business perspective. Some works have focused on specific aspects, such as smart city applications [15], or security [16], or data analytics capabilities [17], across various IoT layers, including cloud services.

While the existing literature offers valuable insights into specific facets of IoT Cloud Platforms, a significant gap remains concerning a comprehensive, feature-driven, and quantitatively scored comparison of a broad range of leading platforms. Many previous reviews either fulfill the following:

- Focus on older platform versions or a smaller, less representative set of platforms, often lacking up-to-date information on the rapidly evolving feature sets [7,14,22].
- Provide high-level qualitative descriptions without granular and comparable metrics for specific features, making direct and objective comparisons challenging [18,19].
- Lack a systematic scoring methodology that allows direct comparison across multiple crucial criteria, often relying on more general comparative discussions rather than a structured quantitative assessment [10,20].
- Do not incorporate a practical decision-support mechanism to assist users in applying the evaluation findings to their unique requirements.

This manuscript aims to bridge these gaps by presenting a detailed evaluation of twelve prominent IoT Cloud Platforms against nine critical, pre-defined features, quantitatively scored based on their capabilities at the time of our research. Our approach not only provides a static comparative snapshot but also introduces a novel interactive decision-making tool. This tool empowers users to dynamically prioritize features based on their specific project needs, thereby transforming a complex multi-criteria decision problem into a personalized, data-driven recommendation. This comprehensive review and accompanying decision-making tool represent a significant contribution to the body of knowledge, offering an up-to-date and practical guide for navigating the complex landscape of IoT Cloud Platforms.

## 3. Methodology

This section details the systematic approach employed for the comprehensive evaluation of IoT Cloud Platforms. To ensure the rigor, objectivity, and reproducibility of our findings, a structured methodology was developed that includes the careful selection of platforms for in-depth analysis, the identification and precise definition of key evaluation features, and the implementation of a robust scoring mechanism. This methodical framework supports the comparative analysis presented in this paper and forms the basis for the subsequent development of our decision-making tool.

### 3.1. Platform Selection Criteria

The selection of IoT Cloud Platforms for in-depth evaluation is a critical initial step, aiming to balance representativeness across the diverse market landscape with practical feasibility for detailed analysis. Our selection process was guided by a multi-faceted approach, prioritizing platforms that demonstrate significant market presence, broad feature sets, and relevance to a wide array of IoT use cases at the time of our research. The twelve platforms ultimately selected for this comprehensive review are: Arduino IoT Cloud, AWS IoT Core, Blynk, Bosch IoT Suite, IoT on Google Cloud Platform, Microsoft Azure IoT Hub, Oracle IoT Cloud Service, Particle IoT Platform, Siemens Insights Hub, ThingsBoard, ThingSpeak, and ThingWorx.

The specific criteria considered for inclusion were as follows:

- **Market Dominance and Popularity:** Platforms from major cloud providers (e.g., AWS, Google, Microsoft, and Oracle) were included due to their extensive ecosystems, significant market share, and widespread adoption across various industries [6,25]. These platforms often serve as benchmarks for feature completeness and scalability.
- **Comprehensive Feature Set:** Priority was given to platforms offering a broad spectrum of IoT functionalities, including device connectivity, data acquisition, processing, analytics, security, and application enablement. This ensured a robust basis for our feature-driven evaluation.
- **Enterprise and Industrial Focus:** Platforms with strong enterprise-grade offerings and those specifically tailored for Industrial Internet of Things (IIoT) applications (e.g., Siemens Insights Hub, Bosch IoT Suite, and ThingWorx) were included to cover the needs of large-scale, mission-critical deployments.
- **Developer Accessibility and Niche Focus:** To provide a balanced perspective beyond large enterprise solutions, platforms known for their developer-friendly environments, specific hardware integration, or niche community popularity (e.g., Arduino IoT Cloud, Particle IoT Platform, Blynk, ThingsBoard, and ThingSpeak) were also considered. These represent viable alternatives for smaller projects, prototyping, or specific vertical applications.
- **Global Reach and Regional Relevance:** The selected platforms demonstrate a global footprint, ensuring the applicability of our evaluation to an international audience while also considering their relevance in key regional markets.
- **Publicly Available Information and Documentation:** While some insights required hands-on exploration, a foundational criterion was the availability of sufficient public documentation, pricing models, and developer resources to enable a consistent and comparable evaluation across all chosen platforms.

This curated selection of platforms provides a representative cross-section of the IoT cloud landscape, encompassing hyperscale providers, industrial specialists, and developer-centric solutions, thus offering a broad and relevant basis for our feature-driven comparative analysis.

### 3.2. Feature Identification and Definition

The efficacy of any comprehensive evaluation is based on the precise identification and clear definition of the criteria against which subjects are evaluated. In the context of IoT Cloud Platforms, the selection of relevant features is paramount as it directly influences the applicability and depth of the comparative analysis. To ensure the evaluation was both comprehensive and relevant to real-world applications, our criteria for feature selection were multifaceted. Our identification of the nine key features was an iterative process, informed by a review of academic literature, analysis of industry reports, examination of vendor documentation, and consideration of common pain points and requirements expressed by IoT practitioners. The goal was to establish a set of features that are universally applicable to diverse IoT deployments, critical to platform performance and usability, and distinct enough to allow meaningful differentiation.

The nine key features identified for the evaluation of IoT Cloud Platforms, along with their significance, are defined as follows:

- **Security:** Security is arguably the most critical aspect of any IoT deployment, encompassing device authentication and authorization, data encryption (at rest and in transit), secure communication protocols, vulnerability management, and access control [26,27,28,29]. Robust security features are fundamental to protecting sensitive data, maintaining system integrity, and preventing unauthorized access or attacks that could have severe real-world consequences.
- **Scalability and Performance:** IoT deployments often involve millions or billions of devices generating vast amounts of data. A platform’s ability to scale horizontally and vertically [30], manage high data throughput, ensure low latency, and maintain high availability under fluctuating load conditions is vital for supporting growing ecosystems and ensuring reliable service delivery.
- **Interoperability:** The IoT ecosystem is inherently heterogeneous, comprising devices, sensors, and systems from numerous vendors utilizing diverse communication protocols (e.g., MQTT, AMQP, CoAP, and HTTP). A platform’s interoperability [31,32], reflected in its support for various protocols, open APIs, and integration capabilities with third-party systems and legacy infrastructure, is crucial for fostering flexible, vendor-agnostic solutions and avoiding vendor lock-in.
- **Data Analytics and AI/ML Integration:** Raw IoT data hold limited value without processing and analysis. This feature assesses a platform’s capabilities for real-time data streaming analytics [33], historical batch processing, machine learning (ML) model training and inference [34,35], and seamless integration with data visualization tools [36]. These capabilities are essential for extracting actionable insights, predicting failures, optimizing operations, and enabling intelligent automation.
- **Edge Computing Support:** Edge computing pushes computation and data processing closer to the data source (devices), reducing latency, conserving bandwidth, and enabling offline operations. Evaluating this feature involves assessing a platform’s support for edge device management, deployment of compute capabilities to the edge, local data processing, and synchronization with the cloud. It is particularly crucial for applications requiring rapid response times or operating in disconnected environments [37].
- **Pricing Models and Cost-effectiveness:** The financial implications of an IoT platform selection are significant for any organization. This feature evaluates the clarity, flexibility, and predictability of pricing models (e.g., pay-as-you-go, tiered, and enterprise agreements), as well as the overall cost-effectiveness for different scales and types of deployments, including potential hidden costs or egress fees [38,39].
- **Developer Tools and SDK Support:** The ease with which developers can build, deploy, and manage IoT solutions directly impacts time-to-market and development costs. This feature considers the availability and quality of Software Development Kits (SDKs) for various programming languages, intuitive development environments, comprehensive documentation, tutorials, and the vibrancy of community support. Research in software engineering for the Internet of Things highlights how robust development tools, platforms, and programming models are essential for efficient application creation and management, ultimately simplifying the development process [40]. Furthermore, analyses of developer discussions on platforms like Stack Overflow underscore the importance of comprehensive documentation and strong community support for mitigating development challenges [41].
- **Compliance and Standards:** Adherence to industry-specific regulations (e.g., GDPR and HIPAA) and international standards (e.g., ISO 27001) is paramount for legal compliance, data governance, and establishing trust, especially in sensitive sectors. This feature assesses a platform’s certifications, audit reports, data residency options, and commitment to industry best practices [42].
- **OTA Update Capabilities:** The ability to remotely update device firmware and software Over-The-Air is crucial for patching security vulnerabilities, deploying new features, and correcting bugs efficiently across large fleets of deployed devices [43,44]. This feature evaluates the robustness, reliability, security, and flexibility of a platform’s OTA update mechanisms.

This carefully defined set of features allows for a systematic and thorough evaluation of each IoT Cloud Platform’s strengths and weaknesses and serves as the foundation for our comparative analysis.

### 3.3. Scoring Mechanism

To provide a clear, comparable, and actionable evaluation of the identified IoT Cloud Platforms, a standardized quantitative scoring mechanism was developed. Each of the twelve selected platforms was evaluated against the nine defined features using a Likert-type 1–10 scoring scale [45], where 1 represents minimal or no support for a given feature, and 10 signifies exceptional, comprehensive, and mature support.

The choice of a Likert-type scale of 1–10 was deliberate, offering several advantages:

- **Granularity:** It provides sufficient granularity to distinguish nuanced differences in feature implementation and maturity across platforms, avoiding the limitations of coarser scales (e.g., 1–5).
- **Intuitiveness:** The scale is widely understood and intuitive, facilitating easy interpretation by a diverse audience of researchers and practitioners.
- **Differentiation:** It allows for a broader spectrum of scoring, enabling a more precise differentiation between platforms that might offer similar, but not identical, levels of support for a given feature.

The process for assigning scores to each platform for every feature involved a rigorous, multi-pronged approach conducted during the research period. This process combined the following:

- **Public Documentation Analysis:** An extensive review of official documentation, white papers, technical specifications, and API references was conducted to understand the stated capabilities, limitations, and architectural design relevant to each feature.
- **Vendor Materials and Industry Reports:** Analysis of vendor-provided marketing materials, solution briefs, case studies, and third-party industry analyst reports (e.g., Gartner Magic Quadrant [46], Forrester Wave [47]) provided insights into platform positioning, strengths, and typical use cases.
- **Trial Account Exploration and Hands-on Testing:** Where feasible, free-tier accounts or trial access were utilized to gain practical experience with platform user interfaces (UIs), developer tools, SDKs and to test basic functionalities related to connectivity, data ingestion, and simple analytics. This allowed for verification of documented claims and assessment of user-friendliness.
- **Expert Judgment and Comparative Benchmarking:** Expert judgment was provided by the authors, who collectively possess over 10 years of experience in IoT architecture, cloud computing, and data security. A structured consensus-building protocol was followed to ensure objectivity and mitigate potential bias [48]. Initially, each author independently assigned preliminary scores for each platform. The inter-rater agreement was then quantitatively assessed using Krippendorff’s Alpha (ordinal data) for each feature group, with resulting Alpha values ranging from 0.72 to 0.88, indicating substantial to almost perfect agreement. Any scoring discrepancies were then subjected to rigorous discussion and cross-validation, where authors justified their ratings based on evidence from documentation, hands-on testing, and comparative benchmarks. The final scores were assigned only after a consensus was reached for every data point, representing a synthesized and thoroughly vetted assessment from our entire research team. This process involved comparing how each platform addressed a specific feature relative to its competitors and against a perceived ideal or industry best practice for that feature. For instance, a platform’s security score considered not just the presence of features like Multi-Factor Authentication (MFA) but also their ease of implementation, integration with other services, and compliance certifications.

It is crucial to emphasize that the scores assigned in this evaluation reflect the state of each platform’s features and capabilities at the time of our research. The IoT Cloud Platform landscape is highly dynamic, with vendors continuously releasing updates, new services, and revising pricing models. Therefore, while this evaluation provides a comprehensive snapshot and robust comparative framework for its time, ongoing developments may alter specific scores in the future. This dynamism underscores the utility of our decision-making tool, which allows users to apply custom weights to these scores, adapting the evaluation to their current priorities and the evolving market.

## 4. Evaluated IoT Cloud Platforms

This section provides a concise overview of the twelve IoT Cloud Platforms subjected to detailed evaluation in this manuscript. Each platform represents a significant offering within the IoT landscape, catering to diverse application needs and target audiences. These are the following:

- **Arduino IoT Cloud** provides a simplified path for Arduino users to connect their projects to the cloud, offering device management, data visualization, and remote control capabilities, ideally suited for hobbyists, makers, and educational projects.
- **AWS IoT Core** is a comprehensive, highly scalable platform designed for secure device connectivity and data management, geared towards enterprise-level IoT solutions.
- **Blynk** distinguishes itself as a user-friendly, low-code platform ideal for rapid prototyping and smaller-scale IoT projects, often favored by hobbyists and educators.
- **Bosch IoT Suite** focuses on industrial applications, offering a robust suite of services for device management, data analytics, and connected manufacturing.
- **IoT on Google Cloud Platform** integrates seamlessly with Google’s broader cloud ecosystem, emphasizing data analytics, machine learning, and scalability for large-scale deployments.
- **Microsoft Azure IoT Hub** provides a fully managed service for secure and reliable bi-directional communication with millions of IoT devices, appealing to a broad range of industries and use cases.
- **Oracle IoT Cloud Service** focuses on enterprise-level IoT solutions, providing a comprehensive suite of services for device management, data analytics, and application enablement.
- **Particle IoT Platform** offers an end-to-end solution for prototyping, deploying, and managing cellular-connected IoT devices, popular among hardware startups and product developers.
- **Siemens Insights Hub** is an Industrial IoT platform built on open standards, focusing on digital transformation in manufacturing and related industries.
- **ThingsBoard** is an open-source IoT platform that enables device management, data collection, processing, and visualization with support for rule-based event handling. It is well suited for custom IoT solutions in both industrial and commercial contexts, offering deployment flexibility across on-premise and cloud environments.
- **ThingSpeak** is a MATLAB-powered IoT analytics platform primarily used for academic and research purposes. It enables real-time data collection, storage, analysis, and visualization from connected devices, with easy integration into the MathWorks ecosystem.
- **ThingWorx** provides a comprehensive application enablement platform, allowing developers to rapidly build and deploy complex IoT applications, primarily targeting industrial and service-oriented use cases.

Based on their features and target audiences, these platforms can be grouped into several key user profiles. The hobbyist and educational communities are primarily served by platforms like Arduino IoT Cloud, Blynk, and ThingSpeak, which offer simplified interfaces and are often integrated with specific hardware or software ecosystems. Startups and developers focused on rapid prototyping and deployment might gravitate towards Particle IoT Platform and ThingsBoard, which provide flexible, end-to-end solutions. For enterprise-level deployments, scalability, security, and integration with broader cloud services are paramount; this is the domain of AWS IoT Core, IoT on Google Cloud Platform, and Microsoft Azure IoT Hub. Finally, industrial and specialized applications are the focus of platforms such as Bosch IoT Suite, Siemens Insights Hub, Oracle IoT Cloud Service, and ThingWorx, which offer robust features tailored for manufacturing, asset management, and other business-critical use cases. This categorization helps to contextualize the detailed feature-driven evaluation that follows, allowing readers to quickly identify platforms most relevant to their specific needs.

The following sections will present a detailed feature-driven evaluation of these platforms, enabling a comparative assessment of their capabilities and suitability for various IoT deployments.

### 4.1. Arduino IoT Cloud

Arduino IoT Cloud [49] is a secure and scalable platform developed to connect, monitor, and manage a broad spectrum of IoT devices, particularly within the Arduino [50] and ESP32 [51] ecosystems. Designed for both accessibility and reliability, the platform integrates real-time dashboards, secure provisioning, OTA updates, and robust APIs. It is tailored for individual developers, educational institutions, and increasingly enterprise customers through its dedicated Enterprise plan. The architecture supports various deployment models and includes tools for edge intelligence, device fleet management, and integration with ML platforms such as Edge Impulse [52]. Leveraging Arduino’s long-standing open-source philosophy and the cloud scalability of AWS [53], Arduino IoT Cloud emerges as a flexible and cost-effective alternative to traditional enterprise-grade platforms like Cisco’s IoT Cloud Center [54].

### 4.2. AWS IoT Core

AWS IoT Core [55] is a fully managed Cloud Platform that enables secure connectivity, device management, and data processing for a vast range of IoT devices. As a core component of AWS, it benefits from the platform’s global infrastructure, scalability, and robust security features, making it a popular choice for enterprise-level IoT deployments. At its heart, AWS IoT Core utilizes MQTT, HTTP, and WebSocket protocols for device connectivity, supporting bidirectional communication and allowing for millions of devices to be securely connected and managed. A key feature is its “Things” registry, a centralized repository for managing device identities and metadata. Device shadows, a virtual representation of each device, allow applications to interact with devices even when they are offline, enhancing reliability and responsiveness. Security is paramount in AWS IoT Core, leveraging AWS Identity and Access Management (IAM) for granular access control and offering end-to-end encryption. Device authentication utilizes mutual Transport Layer Security (mTLS) and customizable security policies. Beyond connectivity, AWS IoT Core seamlessly integrates with a suite of AWS services. AWS IoT Analytics provides data processing and analytics capabilities, while AWS IoT Device Management facilitates OTA updates and remote device configuration. Integration with AWS Lambda allows for serverless, event-driven processing of IoT data, enabling real-time insights and automated actions. AWS IoT Core’s pricing model is based on the number of connected devices, the volume of messages exchanged, and the usage of other integrated AWS services. This flexible model allows organizations to scale their IoT deployments cost-effectively. Targeted towards businesses of all sizes, AWS IoT Core is particularly well suited for IIoT, smart home applications, connected vehicles, and asset tracking. Its extensive feature set, robust security, and seamless integration with the broader AWS ecosystem make it a powerful and versatile platform for building and deploying scalable IoT solutions.

### 4.3. Blynk

Blynk [56] is a low-code platform designed for rapid prototyping and deployment of IoT projects, particularly appealing to makers, hobbyists, and educators. Its key differentiator lies in its ease of use and visual drag-and-drop interface, which allows users to create mobile apps to control and monitor connected devices without extensive coding knowledge. Blynk utilizes a simple cloud-based architecture in which devices connect to the Blynk cloud server, and mobile apps interact with the cloud to control those devices. The platform supports a wide range of hardware, including Arduino, Raspberry Pi, ESP8266, and ESP32. It offers libraries and SDKs for seamless integration. Blynk’s visual interface allows users to quickly create customizable dashboards with buttons, sliders, gauges, and other widgets to visualize data and control devices. While offering a free tier for small projects, Blynk operates on a subscription model with varying features and capacity. The platform excels in quick prototyping and Proof-Of-Concept (POC) projects but may face limitations in scalability and customization for large-scale, complex deployments. Security features include device authentication and encryption, although they may not be as robust as those found on enterprise-grade platforms. Blynk’s primary target audience is individuals and small teams seeking a fast and user-friendly way to build and deploy IoT applications. Its ease of use and visual interface make it ideal for educational purposes, hobbyist projects, and rapid prototyping of IoT concepts. Although lacking some of the advanced features of more complex platforms, Blynk provides an excellent entry point into the world of IoT development.

### 4.4. Bosch IoT Suite

The Bosch IoT Suite [57] is a comprehensive industrial-focused IoT platform designed to develop and deploy connected solutions for a range of industries, including manufacturing, logistics, and smart cities. It provides a complete suite of services that include device management, connectivity, data analytics, and application enablement. Key components include the Device Hub for secure device onboarding and management, the Connectivity Hub for reliable communication, and the Analytics Hub for data processing and visualization. The Bosch IoT Suite emphasizes secure and reliable device connectivity, supporting various protocols like MQTT, HTTP, and CoAP. It also offers advanced device management features, including remote configuration, firmware updates, and security management. The platform’s data analytics capabilities allow users to extract valuable insights from IoT data, leveraging features like time-series data storage, real-time analytics, and machine learning integration. The platform supports various pricing models, including pay-as-you-go and subscription-based plans. It is geared towards businesses seeking a robust and scalable IoT platform for industrial applications, offering features like edge computing support, security certifications, and compliance with industry standards. Its target audience includes industrial manufacturers, logistics providers, and smart city developers seeking to improve operational efficiency, optimize processes, and create new revenue streams.

### 4.5. IoT on Google Cloud Platform

Google Cloud IoT Core was a fully managed service designed to enable secure connectivity, device management, and data processing for a vast range of IoT devices. Integrated seamlessly with the broader Google Cloud Platform (GCP), it leveraged GCP’s infrastructure, scalability, and advanced analytics capabilities. While the service was discontinued on 16 August 2023, understanding its former functionality provides valuable context within the landscape of IoT platforms. Historically, Google Cloud IoT Core functioned as a central hub for connecting devices, managing their configurations, and securely transmitting data to GCP. It allowed for bi-directional communication, enabling remote control and updates to deployed devices. A key strength was its integration with other GCP services, such as Cloud Storage, BigQuery, and Cloud Machine Learning Engine. This allowed users to easily store, analyze, and visualize IoT data and build intelligent applications leveraging Google’s powerful AI and ML capabilities. The platform historically supported various communication protocols, including MQTT and HTTP, and offered robust security features, including device authentication, encryption, and access control. It facilitated device registration, provisioning, and management at scale. Google historically promoted its use in areas like asset tracking, predictive maintenance, and connected operations.

While no longer available as a standalone service, the functionality previously offered by Cloud IoT Core is now being transitioned towards alternative GCP solutions [58]. Google recommends utilizing services like Cloud Pub/Sub, Cloud Functions, and Cloud IoT Edge, combined with existing GCP data analytics and machine learning tools, to achieve similar IoT capabilities. These solutions provide greater flexibility and scalability for building custom IoT applications. Understanding the historical role of Google Cloud IoT Core, and the current GCP-recommended alternatives, is crucial for assessing the evolution of IoT platforms and the continued availability of robust solutions within the Google Cloud ecosystem. This section serves as a historical overview of a previously significant offering in the IoT landscape.

### 4.6. Microsoft Azure IoT Hub

Microsoft Azure IoT Hub [59] is a fully managed cloud service that enables secure and reliable bi-directional communication with millions of IoT devices. Integrated seamlessly with the broader Azure Cloud Platform, it leverages Azure’s infrastructure, scalability, and advanced analytics capabilities. Key components include Device Provisioning Service for secure device onboarding, IoT Hub for device connectivity, and Stream Analytics for real-time data processing. A core strength of Azure IoT Hub is its integration with other Azure services, such as Azure Storage, Azure Data Lake Analytics, and Azure Machine Learning. This allows users to easily store, analyze, and visualize IoT data, and build intelligent applications. It also offers robust security features, including device authentication, encryption, and access control. The platform supports various communication protocols, including MQTT, AMQP, and HTTP. Azure IoT Hub targets a broad range of industries, including manufacturing, retail, and healthcare, offering features like edge computing support, scalability, and integration with other Azure services. Its pricing model is based on device connectivity and data usage, providing a flexible and cost-effective solution for IoT deployments of all sizes.

### 4.7. Oracle IoT Cloud Service

Oracle IoT Cloud Service [60] is a comprehensive, managed, cloud-based Platform-as-a-Service (PaaS) designed to connect, manage, and analyze data from a vast range of IoT devices. Built on Oracle’s cloud infrastructure, this PaaS offers a complete suite of services for device management, data analytics, and application enablement. Core components include Device Management for device onboarding and management, IoT Analytics for data processing and analytics, and IoT Applications for application development. A key differentiator of Oracle IoT Cloud Service is its integration with Oracle’s broader cloud ecosystem, including Oracle Database, Oracle Analytics Cloud, and Oracle Applications. This allows users to leverage existing Oracle investments and expertise to build comprehensive IoT solutions. The platform supports various communication protocols, including MQTT and HTTP, and offers robust security features, including device authentication and encryption. Oracle IoT Cloud Service targets enterprise customers in industries such as manufacturing, logistics, and healthcare, offering features like scalability, security, and integration with other Oracle products. Its pricing model is tailored towards enterprise-level deployments.

### 4.8. Particle IoT Platform

Particle [61] is an end-to-end IoT platform designed to simplify the development and deployment of connected devices. It provides a complete hardware and software ecosystem, including Particle devices, a Cloud Platform, and a mobile app. The platform is particularly well suited for prototyping and deploying cellular-connected IoT devices. Key components include Particle devices, which are low-power, cellular-connected modules, Particle Cloud, a Cloud Platform for device management and data processing, and the Particle Web IDE, a web-based integrated development environment. The platform offers features like OTA firmware updates, device management, and data visualization. Particle targets hardware startups, makers, and product developers seeking a fast and easy way to build and deploy connected devices. Its pricing model is based on device subscriptions and data usage. The platform is known for its ease of use and rapid prototyping capabilities.

### 4.9. Siemens Insights Hub

Siemens Insights Hub (formerly known as MindSphere) [62] is an IIoT platform developed to support digital transformation across industrial sectors. Evolving from Siemens MindSphere, Insights Hub continues to provide a comprehensive suite of tools for connecting, managing, and analyzing data from industrial assets. The platform includes key capabilities such as asset connectivity, edge and cloud-based analytics, and application development. It is tightly integrated with Siemens’ broader industrial automation ecosystem. Originally branded as MindSphere, the platform included components like the MindSphere Industrial IoT operating system, the MindSphere Store, and MindSphere Edge. With the transition to Insights Hub, Siemens emphasizes enhanced usability, improved data insights, and broader ecosystem integration. The platform targets industrial manufacturers, energy providers, and infrastructure operators, with a pricing model oriented towards enterprise-scale deployments.

### 4.10. ThingsBoard

ThingsBoard [63] is an open-source IoT platform designed for device connectivity, data collection, processing, and visualization at scale. It supports multiple connectivity protocols including MQTT, HTTP, and CoAP, making it suitable for a wide range of IoT devices and use cases. The platform features a rule engine for real-time data processing and complex event handling, enabling responsive automation and alerting across diverse applications. ThingsBoard provides a flexible deployment model—available as a cloud-hosted solution or on-premise installation—thus addressing both enterprise and private infrastructure needs. Its architecture separates device management, telemetry data ingestion, and visualization layers, promoting scalability and modular customization. The integrated dashboard builder allows users to create interactive, real-time visualizations and control interfaces. Role-based access control and multi-tenancy support make it a viable option for service providers and industrial system integrators. ThingsBoard also includes OTA update mechanisms, RESTful and RPC APIs, and edge computing capabilities through its ThingsBoard Edge component, which supports distributed deployments closer to the data source. Widely used in smart agriculture, utilities, smart cities, and industrial automation, ThingsBoard stands out for its rich feature set, extensibility, and cost-effectiveness, especially in scenarios where open-source flexibility and full-stack control are critical.

### 4.11. ThingSpeak

ThingSpeak [64] is an IoT analytics platform developed by MathWorks, designed primarily for time-series data collection, analysis, and visualization. Built with seamless integration into the MATLAB [65] environment, it empowers users to perform advanced data processing and algorithm development directly within the platform. ThingSpeak supports HTTP-based communication and is optimized for real-time streaming of sensor data from devices such as Arduino, Raspberry Pi, and ESP8266. The platform allows users to create public or private channels to organize and store device data, with automatic plotting tools for visual analytics. ThingSpeak’s unique value proposition lies in its built-in MATLAB analytics, enabling applications such as anomaly detection, predictive modeling, and energy forecasting without requiring external data pipelines. Educational institutions and researchers frequently adopt ThingSpeak for its accessibility, simplicity, and compatibility with low-cost hardware. While not designed for large-scale or mission-critical deployments, it remains an ideal solution for academic experiments, prototyping, and data-driven learning. With its cloud-based SaaS model, minimal setup complexity, and strong emphasis on data analytics, ThingSpeak addresses a niche in the IoT ecosystem that prioritizes computation and experimentation over industrial scale and integration.

### 4.12. ThingWorx

ThingWorx [66] is an Industrial IoT platform designed to help companies connect, analyze, and act on data from their industrial assets. It provides a comprehensive suite of services for application development, data analytics, and augmented reality. Key components include the ThingWorx platform, ThingWorx Analytics, and ThingWorx AR. The platform offers features like rapid application development, connectivity, data analytics, and augmented reality visualization. ThingWorx targets industrial manufacturers, service providers, and product developers. It is known for its rapid application development capabilities and its focus on industrial applications. Its pricing model is tailored towards enterprise-level deployments.

## 5. Feature-Driven Evaluation and Comparative Analysis

This section presents the core of our research, which is a detailed, feature-driven evaluation, and comparative analysis of the twelve selected IoT Cloud Platforms. Based on the rigorously defined methodology in Section 3, we meticulously assess each platform against the nine identified key features. This analysis aims to provide a granular understanding of each platform’s strengths and weaknesses, offering a comprehensive comparative perspective that is often missing in general market overviews. The insights derived from this evaluation are quantitatively expressed through our 1–10 scoring mechanism, culminating in a comprehensive table that serves as a tangible summary of our findings.

### 5.1. Detailed Feature Analysis

Building upon the foundational overview presented in the previous section, this subsection provides a granular examination of each of the nine identified key features. For each feature, we first discuss its general importance within the IoT ecosystem, highlighting why it stands as a crucial consideration for platform selection. Subsequently, for each of the twelve evaluated IoT Cloud Platforms, we provide a concise analysis of their specific strengths and weaknesses regarding that particular feature. This detailed assessment culminates in a clear justification for the assigned score (1–10), providing a transparent and evidence-based rationale for our quantitative evaluation.

#### 5.1.1. Security

Security is paramount in IoT ecosystems, directly impacting the trustworthiness and resilience of connected solutions. A comprehensive security posture must safeguard data Confidentiality, Integrity, and Availability (the CIA triad) [67]. While a full evaluation would involve in-depth threat modeling using frameworks like STRIDE and vulnerability scoring with systems like CVSS, such an analysis is a study in itself. Therefore, for the purposes of this comparative review, our evaluation focuses on two foundational and directly comparable pillars of platform security: the robustness of cryptographic protocols to protect data in transit and the diversity of authentication mechanisms to ensure secure device and user access. The strength of these fundamental features is a critical initial determinant in a platform’s suitability for secure IoT applications.

Table 1 provides a comparative overview of the security features offered by the evaluated IoT Cloud Platforms, specifically focusing on their cryptographic protocols and authentication mechanisms.

Discussion per Platform:

- **Arduino IoT Cloud** supports secure communication via TLS 1.2, implemented through the BearSSL library [68] for constrained microcontrollers. Device-level authentication is enabled using X.509 certificates, which are securely stored in hardware secure elements (ATECC608) embedded on boards like the MKR and Portenta series [69]. All data transmitted between devices and the cloud are end-to-end encrypted. Arduino IoT Cloud’s backend is hosted on AWS and is ISO/IEC 27001 certified [70], indicating compliance with globally recognized information security management standards. Additionally, user accounts support Two-Factor Authentication (2FA) [71] to enhance login security. These mechanisms ensure Arduino IoT Cloud provides strong, enterprise-ready security capabilities, resulting in a security score of 8 out of 10 due to its comprehensive approach and adherence to industry best practices.
- **AWS IoT Core** demonstrates robust security by supporting the latest TLS versions (1.2 and 1.3) and offering a comprehensive suite of authentication mechanisms. Its support for X.509 certificates, AWS credentials, and various identity management options provides flexibility and strong security posture, suitable for diverse enterprise-grade deployments. The ability to integrate with AWS’s broader security services further enhances its offering, leading to a score 10 out of 10.
- **Blynk** utilizes modern TLS versions for secure communication. However, its primary reliance on OAuth for authentication, while generally secure for web applications, might offer fewer granular device-level control options compared to industrial-grade platforms. The absence of more diverse or hardware-based authentication mechanisms contributes to a slightly lower score of 5 out of 10 for more demanding IoT security requirements.
- **Bosch IoT Suite** leverages TLS 1.2 and 1.3, aligning with current security standards. Its support for OAuth2 and X.509 certificates provides a solid foundation for secure device and application authentication. The inclusion of API keys adds another layer for programmatic access control. This combination of features warrants a strong score of 8.
- **IoT on Google Cloud Platform** supports TLS 1.2 and 1.3 for secure communication. While it leverages Google Cloud’s robust underlying security infrastructure, the explicit details of its authentication mechanisms are not as broadly advertised or easily configurable for specialized IoT devices as some competitors. It receives 10 out of 10, reflecting its strong core security, but with potential for more explicit IoT-centric authentication options.
- **Microsoft Azure IoT Hub** stands out with support for TLS 1.2 and the upcoming TLS 1.3. Its extensive range of authentication mechanisms, including X.509 certificates, Trusted Platform Module, and symmetric keys, offers strong device identity and authentication capabilities, critical for large-scale IoT deployments. The platform’s emphasis on device-level security contributes to its high score 10 out of 10.
- **Oracle IoT Cloud Service** utilizes TLS 1.2 and 1.3 for secure communication. Its reliance on REST resources over HTTP and OAuth for authentication provides a standard and widely accepted approach to security. While effective, the depth of specialized IoT security features might not be as extensive as some dedicated IoT platforms, resulting in a score of 8.
- **Particle IoT Platform** employs DTLS over UDP for reliable and secure datagram communication, alongside AES over TCP, demonstrating a nuanced approach to diverse connectivity needs. Its authentication mechanisms, including Postman, OAuth, and access tokens, offer flexibility for developers. The overall protocol and authentication list is less robust. The inclusion of “Username/Password” as a primary method is a weaker security practice compared to certificate-based systems. The combination of secure protocols and varied authentication methods earns it a score 7 out of 10.
- **Siemens Insights Hub** offers solid authentication with OAuth2 and X.509 certificates. However, its score is lowered by the lack of stated support for TLS 1.3 and the continued presence of older, less secure TLS versions (1.0 and 1.1). Compared to platforms with a broader array of device-specific authentication options or more advanced threat intelligence integrations, it receives a score of 6 out of 10.
- **ThingsBoard** provides a robust and flexible security framework, supporting standard cryptographic protocols like TLS and a variety of authentication mechanisms including basic authentication, X.509 certificates, JWT tokens, and multi-factor authentication options. This comprehensive approach to security, combined with its open-source nature allowing for transparency and community review, makes it a strong contender for secure IoT deployments. This makes ThingsBoard a highly secure and adaptable platform, earning it a score of 8 out of 10 for its robust security features.
- **ThingSpeak** offers essential security features for IoT data transmission, primarily utilizing TLS 1.2 for secure communication over HTTP and MQTT. Its authentication largely revolves around API keys, which are straightforward for quick prototyping and educational purposes. While functional for many applications, the reliance solely on API keys for device authentication may be considered less sophisticated than platforms offering X.509 certificates or OAuth for larger-scale, enterprise-grade deployments requiring stringent identity management. This makes ThingSpeak a convenient platform for rapid IoT prototyping with basic security, earning it a score of 6 out of 10 for its approachable security implementation.
- **ThingWorx** supports SSL, TLS, and AES for secure communication. Its authentication methods, including username/password, API keys, and SSO, provide standard enterprise security features. While these are adequate for many applications, the absence of more advanced device-level hardware-backed authentication or specialized IoT security frameworks limits its score to 7.

#### 5.1.2. Scalability and Performance

Scalability and performance are fundamental considerations for any IoT Cloud Platform, directly influencing its ability to handle growing demands and process data efficiently. Scalability refers to a system’s capacity to gracefully accommodate an increasing number of connected devices, message volumes, and data processing requirements without compromising functionality or user experience [94]. A highly scalable platform can expand its resources—such as compute, storage, and networking—to meet demand peaks, ensuring uninterrupted service. Performance, on the other hand, quantifies the efficiency with which a system processes data and executes operations. This includes metrics, such as message throughput (messages per second), latency (delay in data transmission), and processing speed, for analytics tasks. In the context of IoT, where billions of devices can generate vast streams of data, superior scalability, and performance are essential for real-time analytics, critical decision-making, and maintaining operational continuity.

Table 2 summarizes the scalability and performance characteristics of the evaluated IoT Cloud Platforms based on available documentation and industry insights.

Discussion per Platform:

- **Arduino IoT Cloud** supports a wide variety of hardware, including Arduino (Nano, MKR, Nano, Nicla, Opta, UNO, and Portenta), ESP32/ESP8266-based and LoRaWAN devices [69]. Its cloud backend, hosted on AWS [95], enables scalable communication infrastructure for device fleets. The Arduino IoT Cloud Business (Enterprise) plan [96] adds tools for device group management, organization-wide dashboards, role-based access control, and unlimited data ingestion, making it viable for industrial or commercial applications. While specific throughput or latency figures are not disclosed, the inherited reliability and elasticity of AWS hosting imply robust performance at scale. Overall, the comprehensive feature set and strong AWS foundation earn this platform a solid 8 out of 10 for its potential in IoT deployments.
- **AWS IoT Core** is built on Amazon’s highly scalable cloud infrastructure, designed to handle billions of devices and trillions of messages. Its default throughput of 500 messages per second, with regional variations, demonstrates a robust capacity for high-volume data ingestion. The ability to scale resources dynamically and its global reach positions it as a top-tier platform for large-scale deployments, earning it a score of 10.
- **Blynk** is primarily designed for rapid prototyping and small-to-medium scale personal or hobbyist projects. Its stated limit of 50 requests per device per second indicates a more constrained performance ceiling compared to enterprise-grade platforms. While sufficient for its target audience, this limitation reduces its overall scalability for industrial or large-scale consumer IoT applications, leading to a score of 6.
- **Bosch IoT Suite** offers moderate scalability, with its standard plan supporting up to 1 billion messages per tenant per month. This capacity is suitable for a significant range of industrial IoT applications. The platform’s focus on enterprise solutions suggests a design that can accommodate substantial growth, justifying a score of 8.
- **IoT on Google Cloud Platform** leverages Google’s global, highly scalable infrastructure. Its documented ability to handle 100 messages per second per device indicates strong performance for individual device communication. Given Google Cloud’s extensive capabilities for data processing and analytics at scale, the platform is well suited for large deployments, resulting in a score of 9.
- **Microsoft Azure IoT Hub** is a highly scalable solution within the Azure ecosystem, capable of handling millions of devices and massive message volumes. Its capacity of 5000 send operations per minute per unit demonstrates robust performance for various IoT scenarios, from device-to-cloud to cloud-to-device communication. This strong performance and scalability earn it a score of 9.
- **Oracle IoT Cloud Service** is designed for enterprise-level IoT deployments, leveraging Oracle’s cloud infrastructure for high scalability. While explicit performance metrics are not always readily disclosed, the platform’s focus on large-scale industrial and business applications suggests significant capacity. Its positioning within Oracle’s enterprise ecosystem supports a score of 8.
- **Particle IoT Platform** offers low scalability, with a noted limitation of 1 event per second per device on certain plans. While suitable for many connected product applications and prototyping, this limitation indicates that extremely high-frequency data streams from numerous devices might require architectural adjustments or higher-tier plans. Its ease of use and focus on hardware integration are strong points, but the performance ceiling leads to a score of 6.
- **Siemens Insights Hub** is engineered for industrial IoT, focusing on large-scale data ingestion and processing for manufacturing and smart infrastructure. Its design explicitly supports thousands of messages per second in enterprise environments, showcasing its capability for demanding industrial applications. This specialized focus contributes to a score of 8.
- **ThingsBoard** is a highly scalable open-source IoT platform known for its robust architecture supporting both cloud and on-premises deployments. Its microservices-based design, coupled with support for distributed message queues like Kafka and Cassandra, allows it to handle millions of devices and high message throughputs without a single point of failure. Performance can be tailored to enterprise needs, with cloud plans offering up to 20,000 messages per second per tenant. This makes ThingsBoard an excellent choice for large-scale, demanding IoT solutions, earning it a score of 9 out of 10.
- **ThingSpeak**, while excellent for prototyping and personal projects, offers more limited scalability and performance compared to enterprise-grade platforms. Its free tier imposes significant restrictions, including a 15 s update interval and a low message limit. While paid plans increase update rates to 1 s and expand message capacity, it is primarily designed for data visualization and analysis with MATLAB, not high-volume, real-time industrial applications. Its ease of use and focus on data analysis are strengths, but its throughput limitations result in a score of 5 out of 10.
- **ThingWorx** is a platform tailored for industrial IoT and digital transformation, designed to support high message rates crucial for enterprise configurations. While specific quantitative metrics vary based on deployment and licensing, its architecture is built to handle the substantial data volumes typical of industrial applications, giving it a score of 8.

#### 5.1.3. Interoperability

Interoperability is a critical feature for any IoT Cloud Platform as it defines its ability to seamlessly integrate and communicate with a diverse ecosystem of devices, applications, and other systems. In the fragmented world of IoT, where there is a multitude of hardware vendors, communication protocols, and data formats, the interoperability of a platform directly impacts its flexibility, ease of deployment, and long-term viability. It ensures that data can flow freely and meaningfully between disparate components, preventing vendor lock-in and enabling the creation of cohesive, end-to-end IoT solutions [109]. This capability is often achieved through the support of open standards, various communication protocols, and robust APIs.

Table 3 provides a comparative overview of the interoperability features of the evaluated IoT Cloud Platforms, specifically focusing on the communication protocols they support for device and application integration.

Discussion per Platform:

- **Arduino IoT Cloud** supports standard IoT protocols such as MQTT and RESTful HTTPS allowing devices to communicate efficiently with the platform. It offers an open REST API, enabling integration with third-party applications, and supports automation through services by either using using Webhooks (IFTTT, Zapier, and Google Services) or by offering native support Amazon Alexa, Node-RED, and Google Home. Devices not natively supported by Arduino libraries can still be integrated through custom firmware or direct API interaction. Though it lacks legacy protocols [129] like CoAP, AMQP, XMPP, and DDS offered by other services, Arduino IoT Cloud provides strong mainstream interoperability, earning it a solid score of 9 out of 10 for its balance of features, ease of use, and modern connectivity options.
- **AWS IoT Core** demonstrates strong interoperability through its support for widely adopted IoT protocols like MQTT, HTTPS, and WebSockets. This broad protocol support allows for flexible integration with a wide range of devices and applications. Its extensive ecosystem of AWS services further enhances its ability to connect with various data sources and downstream systems, earning it a score of 8.
- **Blynk** primarily relies on its proprietary Blynk Protocol for efficient communication within its ecosystem. While it also offers HTTPS and MQTT support, the emphasis on its custom protocol can sometimes limit its seamless integration with devices and systems outside the immediate Blynk environment. This slightly less universal protocol support leads to a score of 7.
- **Bosch IoT Suite** offers good interoperability by supporting standard protocols such as MQTT, HTTPS, and AMQP. This combination caters to various messaging patterns and ensures compatibility with a broad array of devices and enterprise systems, making it a robust choice for industrial IoT applications. It receives a score of 7.
- **IoT on Google Cloud Platform** offers a robust level of interoperability by supporting standard protocols such as MQTT and HTTP, along with seamless integration into Google Cloud’s broader ecosystem through services like Pub/Sub, Cloud Functions, and BigQuery. Although its native IoT Core service was deprecated, equivalent functionality can still be achieved using Google’s messaging and API infrastructure, with extended protocol support (e.g., LoRaWAN and CoAP) enabled through third-party integrations. This flexibility and the strong API-driven architecture contribute to its high interoperability, earning it a score of 9 out of 10.
- **Microsoft Azure IoT Hub** provides excellent interoperability with support for MQTT, HTTPS, AMQP, and WebSockets. This comprehensive suite of protocols caters to a wide range of device capabilities and application requirements, enabling seamless integration with both constrained devices and advanced enterprise systems. Its broad protocol support contributes to its score of 8.
- **Oracle IoT Cloud Service** offers standard interoperability through its support for MQTT and HTTPS. These fundamental protocols ensure broad compatibility for basic device connectivity and data exchange. While adequate for many enterprise IoT applications, the platform’s protocol diversity is not as extensive as some leading competitors, thus its score of 7.
- **Particle IoT Platform** focuses on seamless integration with its hardware, supporting MQTT and CoAP. While these are efficient for connected devices, its protocol support is more specialized towards its hardware ecosystem. This narrower focus compared to platforms designed for universal interoperability results in a score of 5.
- **Siemens Insights Hub** offers interoperability primarily through MQTT and HTTPS, which are standard for industrial data exchange. Its strength lies in its ability to integrate with industrial automation systems through specific connectors, although its general protocol support is more focused than some hyper-scale cloud providers. It receives a score of 7.
- **ThingsBoard** offers a highly flexible and extensible platform with strong interoperability, supporting a comprehensive suite of standard IoT protocols such as MQTT, HTTP, CoAP, LwM2M, and SNMP, alongside a powerful REST API for integration. Its open-source nature and robust gateway capabilities further enhance its ability to connect various devices and integrate with enterprise systems, making it suitable for complex and diverse IoT deployments. This makes ThingsBoard a very versatile platform for connecting a wide range of devices, earning it a score of 8 out of 10 for its extensive and adaptable interoperability features.
- **ThingSpeak** provides a straightforward and accessible platform, primarily focused on collecting, analyzing, and visualizing IoT data, especially through its strong integration with MATLAB. While it offers essential connectivity via MQTT and HTTP protocols, its interoperability with a broader range of protocols or complex enterprise systems is more limited compared to some other platforms. It excels in simplicity and ease of use for quick prototyping and educational purposes rather than large-scale, multi-protocol deployments. This makes ThingSpeak a solid choice for basic data collection and analysis, earning it a score of 6 out of 10 for its functional but less extensive interoperability.
- **ThingWorx** excels in interoperability, particularly within industrial environments. Beyond standard protocols like MQTT, HTTPS (via AlwaysOn), and WebSockets, it offers native support for industrial protocols such as OPC and SNMP, as well as ODBC and extensive REST APIs. The availability of additional protocols through the PTC Store further broadens its integration capabilities, making it highly versatile for complex industrial IoT solutions, earning it a leading score of 9.

#### 5.1.4. Data Analytics and AI/ML Integration

The immense volume, velocity, and variety of data generated by IoT devices necessitate sophisticated capabilities for data analytics and artificial intelligence/machine learning (AI/ML) integration. These technologies are crucial for transforming raw IoT data into actionable insights, enabling predictive maintenance, anomaly detection, optimized operations, and enhanced decision-making [130]. A robust IoT Cloud Platform must, therefore, provide not only efficient data ingestion but also powerful tools for real-time processing, historical analysis, and seamless integration with advanced AI/ML services. This integration allows organizations to uncover hidden patterns, automate responses, and derive maximum value from their connected assets.

Table 4 presents a comparative analysis of the data analytics and AI/ML integration capabilities offered by the evaluated IoT Cloud Platforms.

Discussion per Platform:

- **Arduino IoT Cloud** platform supports real-time data monitoring, interactive graphical dashboard creation, and alert-driven automation by leveraging customizable widgets and user-defined interfaces. Historical data can be viewed and exported for further analysis. For machine learning capabilities, the ML tool Addon, powered by Edge Impulse, is available, while on the device level, boards like the Arduino Nano 33 BLE Sense support on-device ML inference using TensorFlow Lite or Google LiteRT. While the platform does not include built-in ML pipelines, Arduino IoT Cloud can be integrated with external ML systems (e.g., AWS SageMaker, Google Colab, and Microsoft Azure ML) via webhooks, REST APIs, or MQTT, making it suitable for intelligent IoT applications. Overall, considering its balance of features, ease of integration, and potential for expansion, we assign the Arduino IoT Cloud platform a score of 7 out of 10.
- **AWS IoT Core** excels in data analytics and AI/ML integration due to its deep ties with the extensive AWS ecosystem. It offers dedicated services like AWS IoT Analytics for specialized IoT data processing and QuickSight for business intelligence. Crucially, its seamless integration with powerful AI/ML services such as AWS SageMaker, Amazon Rekognition, and Amazon Comprehend allows for advanced predictive analytics, machine learning model deployment, and real-time inference at the edge or in the cloud. This comprehensive and integrated approach makes it a leader in this category, earning a score 10 out of 10.
- **Blynk** provides basic data analytics capabilities, primarily focused on real-time monitoring and visualization of sensor data through its intuitive mobile and web applications. While it offers fundamental tools for tracking and displaying IoT data, its native support for advanced statistical analysis or integrated AI/ML model training/deployment is limited. Users would typically need to export data to external platforms for complex AI/ML tasks, resulting in a score of 4.
- **Bosch IoT Suite** offers a solid foundation for data analytics with its Bosch IoT Analytics component, facilitating real-time monitoring, data analysis, and anomaly detection. While it supports integration with Bosch’s own ML solutions and external AI platforms, its native AI/ML capabilities are not as extensively developed or broadly integrated as the hyper-scale cloud providers. This positions it as a capable platform with good potential for specialized industrial use cases, but a score of 7 reflects its current offerings compared to others.
- **IoT on Google Cloud Platform** leverages the formidable data analytics and AI/ML capabilities of the broader Google Cloud Platform. It seamlessly integrates with BigQuery for large-scale data warehousing and SQL analytics, Dataflow for real-time data processing, and crucially, Google AI Platform (now Vertex AI) for advanced ML model training, deployment, and inference. This native and deep integration with industry-leading AI/ML services makes it a powerhouse for data-driven IoT applications, earning a score 10 out of 10.
- **Microsoft Azure IoT Hub** provides excellent integration with Azure’s comprehensive suite of analytics and AI/ML services. Users can leverage Azure Stream Analytics for real-time data processing, Power BI for interactive dashboards and reporting, and critically, Azure Machine Learning for building, training, and deploying ML models. Azure Cognitive Services further enhances its capabilities for vision, speech, and language AI. This robust and integrated offering makes it a strong contender, earning a score 10 out of 10.
- **Oracle IoT Cloud Service** provides Oracle Analytics for IoT, which includes built-in machine-learning-based anomaly detection and predictive analytics features tailored for IoT data. It also integrates with Oracle Machine Learning services, allowing users to develop and deploy custom predictive models. While capable, its ecosystem of AI/ML tools is generally less extensive or diverse than the offerings from the leading hyperscalers, leading to a score of 7.
- **Particle IoT Platform** offers basic analytics and visualization tools for real-time monitoring and performance tracking of connected devices. However, its native support for advanced AI/ML functionalities is minimal. Users who require complex machine learning or deep learning capabilities would typically need to export their data to external cloud-based AI/ML platforms for processing, resulting in a score of 5.
- **Siemens Insights Hub** offers comprehensive analytics capabilities through data analytics and various visualization tools, particularly strong for industrial use cases. It integrates with Siemens’ own AI tools and allows for connections to external ML platforms, crucial for predictive maintenance and operational optimization in manufacturing and smart infrastructure. While robust for its domain, its general AI/ML breadth might be slightly less than the major cloud providers for non-industrial specific applications, leading to a score of 8.
- **ThingsBoard** offers robust analytics through its Trendz Analytics module, providing features like real-time monitoring, historical data analysis, anomaly detection, and forecasting. Its AI/ML integration is quite strong, allowing users to leverage custom Python models and connect with various Large Language Models (LLMs) such as OpenAI, Amazon Bedrock, and Google Gemini, which significantly enhances its predictive capabilities and broadens its applicability across different industrial use cases. This comprehensive approach to data analysis and integration of advanced AI tools makes ThingsBoard a powerful platform for complex IoT solutions, earning it a score of 8 out of 10 for its comprehensive analytics and AI/ML capabilities.
- **ThingSpeak**, developed by MathWorks, excels in its native integration with MATLAB, making it particularly strong for users who are already familiar with MATLAB’s powerful analytical and computational capabilities. It provides immediate visualization of data, online processing, and the ability to schedule MATLAB code to run directly on the platform for advanced data manipulation and algorithm deployment. While it might require more coding knowledge (specifically MATLAB) compared to some other platforms for advanced analytics, its direct access to MATLAB’s extensive libraries for machine learning and predictive modeling offers significant potential for sophisticated IoT data analysis. This deep integration with MATLAB, despite a potential higher barrier to entry for some, earns it a score of 7 out of 10 for its analytical prowess and integrated AI/ML capabilities.
- **ThingWorx** provides advanced analytics functionalities via ThingWorx Analytics, enabling real-time insights, anomaly detection, and predictive modeling tailored for industrial IoT. It offers native support for integrating and deploying AI/ML models directly within the platform and can also integrate with external ML platforms. Its focus on providing actionable intelligence for industrial operations makes it a strong performer in its niche, earning a score of 8.

#### 5.1.5. Edge Computing Support

Edge computing represents a transformative paradigm in IoT, fundamentally shifting where data processing and computation occur within a network. Unlike traditional cloud computing, which centralizes processing in remote data centers, edge computing brings these capabilities closer to the data source, at the “edge” of the network [154]. This distributed architecture minimizes latency, reduces bandwidth consumption, enhances data security by processing sensitive information locally, and enables near real-time decision-making without constant reliance on cloud connectivity. For IoT applications, edge computing is crucial for scenarios requiring immediate responses, offline operation, or efficient handling of massive data volumes at the source, such as industrial automation, autonomous vehicles, and smart cities.

Table 5 outlines the edge computing support offered by the evaluated IoT Cloud Platforms, highlighting their key products and capabilities in this domain.

Discussion per Platform:

- **Arduino IoT Cloud** offers edge intelligence primarily through its Arduino PRO-Edge IoT technology [155]. The list of supported hardware includes but is not limited to Portenta [156], Nicla [157], and MKR [158] series, which feature dual-core ARM processors and onboard ML acceleration. These boards enable local computation, reducing latency and cloud dependency. Data can be filtered or processed on the edge before being sent to the cloud. Considering its hardware support and capabilities, the Arduino IoT Cloud’s edge intelligence offering earns a score of 7 out of 10, showing solid potential but with room for expansion in terms of advanced analytics and broader device compatibility.
- **AWS IoT Core** provides exceptional edge computing capabilities through AWS IoT Greengrass. Greengrass allows developers to extend AWS cloud capabilities, such as Lambda functions, machine learning inference, and data processing, directly to edge devices. This enables local execution of logic, data filtering, and real-time responses, even with intermittent connectivity, making it a highly robust solution for complex edge deployments. This comprehensive offering earns it a score of 9.
- **Blynk** offers an intuitive and low-code approach to IoT development, primarily leveraging its cloud platform for device connectivity, mobile app creation, and data visualization. While devices connected to Blynk can execute logic locally, such “edge processing” is predominantly handled by the device’s firmware and microcontroller capabilities, rather than a dedicated edge computing product or framework provided by Blynk. Crucially, the previously available Blynk Legacy Local Server has been discontinued [161] and is no longer supported, meaning there is no officially maintained on-premise server option for the current Blynk IoT platform. Features like OTA updates and device provisioning, facilitated by “Blynk.Edgent” [162], are valuable for managing devices at the edge, but they do not constitute comprehensive edge computing capabilities like local data filtering, complex event processing, or full offline operability at a gateway level. This positions Blynk as highly accessible for quick development and cloud-centric applications but with limited dedicated support for advanced edge computing scenarios. This makes Blynk more suitable for rapid prototyping and cloud-dependent applications, earning it a revised score of 2 out of 10 for its edge computing support.
- **Bosch IoT Suite** provides solid edge computing support through its Bosch IoT Edge Agent and associated Edge Services. These components enable local data aggregation, processing, and filtering on edge devices. This capability is particularly relevant for industrial IoT scenarios where local data handling and real-time operations are crucial, contributing to a score of 7.
- **IoT on Google Cloud Platform** offers strong edge computing support via Google Cloud IoT Edge. This solution allows for the deployment of Google Cloud services, custom logic, and trained AI/ML models directly onto edge devices. This enables powerful local analytics, real-time decision-making, and reduced reliance on constant cloud connectivity, making it a highly capable platform for sophisticated edge deployments. It receives a score of 9.
- **Microsoft Azure IoT Hub** provides excellent edge computing functionalities through Azure IoT Edge. This service allows users to deploy cloud workloads, including AI, analytics, and custom business logic, directly to edge devices. It supports offline capabilities, module management, and secure communication, making it highly versatile for various edge scenarios, from simple data filtering to complex AI inference. This comprehensive offering warrants a score of 9.
- **Oracle IoT Cloud Service** offers edge computing support through its Oracle Roving Edge Infrastructure. This unique offering provides ruggedized, portable edge devices that extend Oracle Cloud services to remote or disconnected environments. While powerful for specific use cases requiring a self-contained, distributed cloud presence, it is a more specialized approach to edge computing compared to software-centric frameworks offered by others. This specialized capability earns it a score of 7.
- **Particle IoT Platform** provides edge computing capabilities with Edge ML, which allows for deploying lightweight machine learning models directly onto Particle microcontrollers. This enables local inference and smart decision-making at the device level, reducing latency and bandwidth usage. While powerful for resource-constrained devices, its scope might be more focused on specific ML applications rather than broader edge orchestration, earning a score of 7.
- **Siemens Insights Hub** offers robust edge computing support tailored for industrial environments through MindConnect for secure data acquisition and Industrial Edge for deploying applications and analytics directly onto industrial automation devices. This integration allows for real-time processing and control at the factory floor, crucial for Operational Technology (OT) convergence. Its strength lies in its industrial-specific edge capabilities, earning a score of 7.
- **ThingsBoard** offers ThingsBoard Edge, a robust and flexible solution for extending IoT data processing and management to the network’s edge. It enables local data storage, real-time analytics, and alarm generation, ensuring critical operations can continue even with unreliable cloud connectivity. Its ability to filter data at the source helps reduce bandwidth consumption and cloud processing costs, making it highly efficient for diverse industrial and enterprise applications. ThingsBoard Edge integrates seamlessly with the main ThingsBoard platform, allowing for synchronized updates and centralized management of distributed edge deployments, thus providing a comprehensive edge-to-cloud solution. This makes ThingsBoard a very strong contender in the edge computing space, earning it a score of 8 out of 10 for its comprehensive and efficient edge capabilities.
- **ThingSpeak** is primarily known as a powerful IoT analytics platform for aggregating, visualizing, and analyzing live data streams in the cloud, particularly with its strong integration with MATLAB. While it is excellent for rapid prototyping and educational purposes, its native support for advanced edge computing capabilities, such as complex local rule processing, offline operation, and extensive device management at the edge, is limited. Edge functionality with ThingSpeak often relies on external hardware (like Raspberry Pi or Arduino) to perform local computations and data filtering before sending data to the cloud. Its strength lies in cloud-based data analysis rather than robust edge infrastructure. This focus on cloud analytics rather than dedicated edge solutions earns ThingSpeak a score of 4 out of 10 for its edge computing support.
- **ThingWorx** provides strong edge computing capabilities with its ThingWorx Edge MicroServer (EMS). EMS facilitates local data collection, processing, and filtering on edge devices, enabling near real-time operations and reducing the need for constant cloud connectivity. This is particularly beneficial for industrial and enterprise applications where immediate insights and actions at the source are critical, earning a score of 7.

#### 5.1.6. Pricing Models and Cost-Effectiveness

The selection of an IoT Cloud Platform is profoundly influenced by its pricing model and overall cost-effectiveness. Unlike a simple per-unit cost, IoT platform pricing can be highly complex, varying based on factors such as the number of connected devices, message volume, data storage, data processing, usage of specialized services (e.g., analytics and AI/ML), and network egress fees. IoT platform providers offer diverse pricing structures designed to align with the varied needs of businesses, from small-scale deployments and home ecosystems to vast enterprise implementations. The true cost-effectiveness of a solution extends beyond the listed price; it encompasses operational efficiency, scalability, ease of deployment, and the alignment of the platform’s features with specific user requirements, ultimately impacting the Total Cost of Ownership (TCO). Understanding these nuances is crucial for strategic financial planning and ensuring the long-term viability of an IoT project.

Table 6 presents a comparative overview of the pricing models and a qualitative assessment of the cost-effectiveness for each of the evaluated IoT Cloud Platforms.

Discussion per Platform:

- **Arduino IoT Cloud** offers a transparent, tiered pricing model [173] that includes a Free Plan for 2 Things (2 devices), Maker Plan (USD 6.99/month for 1 user—25 devices), and a scalable Enterprise Plan, which is available via the AWS Marketplace [53] (USD 5,000/year for 500 devices) and Prototype Plan (USD 240/year for 20 devices). All tiers support secure communication, real-time dashboards, and OTA updates. Arduino’s pricing model is particularly attractive to SMEs and developers especially for prototyping. Considering its accessibility for beginners and scalability for larger projects, the Arduino IoT Cloud pricing receives a score of 6 out of 10.
- **AWS IoT Core** excels in scalability and cost-effectiveness through its flexible pay-as-you-go model. The substantial Free Tier allows extensive testing and small deployments, making it attractive for new ventures. As usage grows, the volume discounts for messages and granular pricing for various integrated services (like S3 or Lambda) ensure that costs remain optimized, preventing overpayment for unused resources. While the complexity of managing numerous interconnected services can require careful attention to avoid unexpected expenses, its robust ecosystem and ability to scale from minimal to massive deployments make it a top choice for enterprises prioritizing flexibility and global reach. Given its high scalability and granular cost control, AWS IoT Core pricing earns a score of 9 out of 10.
- **Blynk** stands out for its simplified, transparent tiered pricing, offering exceptional cost-effectiveness, particularly for small-scale projects, rapid prototyping, and personal or small business applications. Its Free Tier provides enough functionality to get started with basic device management and data streams. The Plus and Pro plans offer clear, predictable costs as device and user counts increase, scaling smoothly for growing needs without significant complexity. The inclusion of features like device templates and historical data retention within these tiers adds significant value, making it a highly attractive option for developers who prioritize ease of use and quick deployment without incurring large, unpredictable costs. For its clear, predictable, and highly cost-effective tiered plans, Blynk receives a score of 6 out of 10.
- The **Bosch IoT Suite** targets industrial and enterprise applications, evidenced by its subscription-based, custom pricing model. While specific details are not publicly listed, implying a focus on bespoke solutions for large-scale deployments, its cost-effectiveness is primarily realized within the Bosch ecosystem. For companies already utilizing Bosch technology, this platform offers predictable costs for managed services and seamless integration. However, the lack of public pricing and likely higher entry barrier may make it less appealing for smaller projects or businesses not already embedded in Bosch’s industrial framework, requiring direct consultation to understand the true investment. Due to its bespoke and less transparent pricing, Bosch IoT Suite is scored 5 out of 10.
- **IoT on Google Cloud Platform**. While Google Cloud IoT Core is being deprecated, Google Cloud’s general IoT strategy revolves around a pay-per-use model for data ingestion and egress. The substantial USD 300 free credit for new users allows extensive exploration of its capabilities, including its robust integrated services like BigQuery, AI Platform, and Pub/Sub. This model provides immense flexibility for scalable deployments, enabling users to grow their IoT infrastructure organically and only pay for the resources consumed. While it demands careful management of costs associated with various integrated services, its ability to support diverse and large-scale applications with granular control over spending makes it a very strong contender for scalable and data-intensive IoT solutions. Recognizing its flexibility, scalability, and free credit offering, IoT on Google Cloud Platform’s pricing earns a score of 9 out of 10.
- **Microsoft Azure IoT Hub** provides a highly cost-effective and versatile platform, suitable for a wide range of deployment sizes. Its tiered pricing (Basic and Standard) based on messages and data transfer, alongside a generous Free Tier, makes it accessible for small-scale projects and rapid development. The clear per-unit pricing for IoT Hubs ensures predictability, while higher tiers support advanced features and increased message quotas for enterprise-grade solutions. Its deep integration with other Azure services simplifies development and operations. This combination of predictable costs, extensive features, and broad accessibility positions Azure IoT Hub as a highly competitive and cost-efficient choice for diverse IoT applications. With its predictable tiers and broad applicability, Microsoft Azure IoT Hub receives a score of 9 out of 10.
- **Oracle IoT Cloud Service** primarily targets enterprises already operating within Oracle’s ecosystem, employing a device-based monthly pricing model with included messages and metered additional services. The lack of readily public specific pricing details necessitates direct consultation, which can be a barrier for smaller or exploratory projects. While potentially effective for organizations deeply integrated with Oracle’s existing enterprise software and cloud infrastructure, its cost structure may be less flexible or comparatively higher for independent projects outside this established ecosystem. Transparency in pricing is lower than many hyperscalers, making it less straightforward for cost estimation without direct engagement. Given its limited pricing transparency and enterprise-specific focus, Oracle IoT Cloud Service is assigned a score of 4 out of 10.
- **Particle IoT Platform** offers a highly integrated hardware and cloud solution, making it particularly cost-effective for startups, developers, and small-to-medium deployments focused on seamless hardware–cloud integration. While specific current pricing often requires custom quotes beyond its Free Tier, its historical plans (like “Developer”) have catered well to smaller scales. The value lies in its streamlined development experience, pre-certified hardware, and unified ecosystem, which can significantly reduce time-to-market and development costs. This integrated approach simplifies connectivity and device management, offering substantial value for projects where rapid deployment and ease of use are critical factors. For its strong hardware–cloud integration and developer-friendly approach, the Particle IoT Platform receives a score of 7 out of 10.
- **Siemens Insights Hub** is positioned as an “Open IoT Operating System” primarily for manufacturing and industrial enterprises. Its subscription-based pricing, which factors in devices, data model complexity, and application usage, is typically custom and not publicly disclosed. This bespoke pricing model and emphasis on specific industrial modules reflect a focus on large-scale, high-value industrial applications, often requiring significant initial investment. While it offers the most economical solution for companies deeply invested in Siemens’ existing industrial technology and infrastructure, its high entry cost and tailored approach make it less suitable or cost-effective for smaller projects or those outside the heavy industrial sector. Reflecting its high entry barrier and industrial focus, Siemens Insights Hub is scored 3 out of 10.
- **ThingsBoard** offers a highly cost-effective hybrid model that appeals to a wide range of users. Its open-source Community Edition provides a completely free solution for self-hosting, ideal for developers and small projects with technical expertise. The Professional Edition (PE) extends this by offering tiered, device-based subscriptions for both cloud and self-managed deployments, providing excellent scalability with predictable pricing. From the affordable Maker plan to enterprise-grade custom solutions, ThingsBoard balances open-source flexibility with commercial support and advanced features. This makes it a very attractive option for those seeking robust IoT platform capabilities without being locked into proprietary systems or incurring prohibitive costs. Considering its open-source flexibility and scalable commercial options, ThingsBoard earns a score of 9 out of 10.
- **ThingSpeak** is a very cost-effective platform, particularly for hobbyists, educational purposes, and small-scale non-commercial projects, primarily due to its generous free service tier. This tier allows up to 3 million messages per year and four channels, with a 15 s update interval, providing ample room for learning and experimentation. For larger home or commercial projects, the Home and Standard licenses offer clear, unit-based pricing with increased message limits, channels, and faster update intervals. Its strong integration with MATLAB analytics adds significant value for users requiring data processing and visualization capabilities, making it an excellent choice for data-centric IoT applications. Given its free tier and clear, scalable pricing for data-focused applications, ThingSpeak receives a score of 7 out of 10.
- **ThingWorx** targets large enterprises and industrial IoT solutions with a subscription-based model where pricing is available only upon request and typically tailored to specific needs. Its focus on significant investment for large deployments suggests it is not designed for small or hobbyist projects. While comparative analyses might place its cost in a similar range to hyperscalers like AWS IoT Core or Azure IoT Hub for extensive deployments, its enterprise-centric approach implies a higher barrier to entry and a more complex procurement process. ThingWorx is best suited for organizations requiring highly customized, industrial-grade IoT solutions with significant dedicated resources. Due to its enterprise-only focus and lack of transparent pricing for smaller users, ThingWorx is assigned a score of 3 out of 10.

#### Use Case: Comparative Cost Analysis for a Medium-Scale IoT Deployment

To provide a practical context for the financial implications of platform selection, a representative use case was modeled to evaluate cost-effectiveness. This scenario envisions a medium-scale deployment consisting of 1,000 IoT devices, each configured to transmit a 100-byte message every 10 min. This transmission frequency equates to 144 messages per device per day, culminating in a total annual data volume of approximately 5 GiB for all participating devices. An investigation of the pricing packages offered by each vendor was conducted to determine the annual operational cost for this specific deployment scenario.

The cost analysis reveals great variations in annual pricing across the spectrum of evaluated platforms. The hyperscaler platforms proved to be the most economical for this use case, with AWS IoT Core offering a notably low annual cost of USD 53, considering only the messaging costs. Both Microsoft Azure IoT Hub and Google Cloud presented competitive and identical pricing at USD 300 per year. In the mid-range, costs varied significantly. Particle’s offerings included a basic plan at USD 3000 and a more advanced plan at USD 6000 annually, while ThingSpeak and ThingsBoard were estimated at USD 3064 and USD 8988 per year, respectively. Blynk’s annual cost was calculated to be USD 7000.

At the higher end of the cost spectrum were platforms primarily targeting large enterprise or industrial applications. Arduino IoT Cloud’s annual cost was estimated at USD 10,000, while Oracle IoT Cloud Service was the most expensive at USD 12,000 per year. For several platforms focused on enterprise solutions, pricing was not listed transparently. Based on available information, an equivalent deployment on the Bosch IoT Suite was estimated to be in the region of USD 5000. For Siemens Insights Hub and ThingWorx, costs are typically determined through custom enterprise quotations and were, therefore, not specified in this analysis. This investigation clearly demonstrates that for identical technical requirements, the financial commitment can differ by orders of magnitude, highlighting that a thorough evaluation of pricing models is a critical factor in determining a platform’s TCO.

#### 5.1.7. Developer Tools and SDK Support

The availability and quality of developer tools and SDKs are pivotal for the efficient and scalable development, rapid deployment, and streamlined management of IoT solutions. These resources significantly impact developer productivity, reduce time-to-market, and simplify the complex task of integrating diverse devices and applications with Cloud Platforms. Comprehensive SDKs, accessible APIs, intuitive development environments, and robust documentation empower developers to connect devices, ingest data, build applications, and leverage advanced platform features effectively. Therefore, the strength of a platform’s developer ecosystem is a critical criterion for selecting the most suitable IoT cloud provider based on specific business requirements and developer preferences.

Table 7 presents a comparative overview of the developer tools and SDK support offered by the evaluated IoT Cloud Platforms.

Discussion per Platform:

- **Arduino IoT Cloud** features a web-based Cloud Editor [187], Arduino CLI [188], and auto-generated device sketches [189] to streamline development. REST APIs and Arduino/C++/JavaScript/Python SDKs enable integration with third-party applications and enterprise systems [190,191,192,193]. Available documentation is extensive and supports both beginner and advanced users, supported by a very active community of developers. Arduino provides a broader appeal across technical proficiency levels compared to its competitors, earning it a solid score of 8 out of 10 for its comprehensive features and accessibility.
- **AWS IoT Core** provides an exceptionally rich set of developer tools and SDKs across various programming languages (Embedded C, C++, Python, JavaScript, and Java) and platforms (mobile SDKs for Android and iOS). Its comprehensive Command Line Interface (CLI) and extensive documentation further simplify development and integration. This wide range of well-maintained and actively supported SDKs significantly accelerates development cycles for diverse IoT applications, leading to a score of 9.
- **Blynk** offers user-friendly SDKs and libraries, primarily focused on simplifying the connection of devices to its platform, notably with its Blynk.Edgent packaged solution and portable C++ libraries for popular microcontrollers like Arduino and ESP32. While excellent for rapid prototyping and app-centric IoT solutions, its SDKs are more tailored to its specific ecosystem, offering less breadth for highly customized enterprise integrations compared to hyper-scale cloud providers. This specialized yet effective support earns it a score of 7.
- **Bosch IoT Suite** supports standard development approaches with Java SDKs and a REST-like HTTP API. Furthermore, its alignment with and contributions to key Eclipse IoT open-source projects like Eclipse Hono, Ditto, Vorto, and hawkBit significantly enhance its interoperability and developer flexibility for building robust industrial IoT solutions. This commitment to open standards and practical SDKs merits a score of 7.
- **IoT on Google Cloud Platform**. Google Cloud IoT Core’s official deprecation as of August 2023 significantly impacts its developer tool and SDK support. While general Google Cloud SDKs and client libraries can still be used to interact with other Google Cloud services that might process IoT data, there is no longer a dedicated, actively developed SDK specifically for IoT device connection and management through a centralized IoT Core service. This fundamental change severely limits its viability for new IoT projects, leading to a low score of 3.
- **Microsoft Azure IoT Hub** provides an excellent developer experience with comprehensive SDKs available in multiple popular languages including C, .NET, Node.js, Java, and Python. It also offers specialized embedded SDKs for various real-time operating systems like Eclipse ThreadX and FreeRTOS, as well as bare metal environments. The extensive Azure CLI and integration with Visual Studio Code further enhance developer productivity, earning it a score of 9.
- **Oracle IoT Cloud Service** provides foundational SDKs in Java, JavaScript, and C Posix, alongside a well-documented REST API for broader integration. It also offers mobile SDKs for Android and iOS, facilitating the development of mobile IoT applications. These tools provide adequate support for enterprise IoT solutions within the Oracle ecosystem, leading to a score of 7.
- **Particle IoT Platform** stands out for its strong developer focus, offering the Particle API JS (JavaScript SDK), comprehensive SDKs for its hardware devices, and API libraries in Node.js and Python. It also provides mobile SDKs for Android and iOS, making it easy to build end-to-end solutions. Its integrated development environment and strong community support further enhance the developer experience, earning it a score of 8.
- **Siemens Insights Hub** offers a robust set of SDKs for common programming languages like Java, Python, and Node.js. It also provides a specialized Fleet Manager Plugin SDK and the Open Edge Device Kit, which are crucial for industrial IoT applications. Its strong API documentation and focus on enabling digital twin creation and application development for industrial use cases make it highly functional for its target audience, meriting a score of 8.
- **ThingsBoard** offers a robust and highly flexible open-source IoT platform with extensive developer tools and SDK support. It provides comprehensive APIs across various protocols like MQTT, CoAP, HTTP, LwM2M, and SNMP, enabling broad device connectivity. Developers benefit from readily available client libraries in popular languages like Python, Java, and Dart, alongside a dedicated Arduino SDK for embedded development. The platform also supports mobile application development through Flutter, enhancing its versatility for custom solutions. This makes ThingsBoard a powerful choice for both rapid prototyping and scalable enterprise deployments, earning it a score of 9 out of 10 for its rich and diverse developer ecosystem.
- **ThingSpeak**, developed by MathWorks, positions itself as an IoT analytics platform, excelling in data visualization and analysis, particularly with its integrated MATLAB engine. While it provides essential REST and MQTT APIs for device communication, its primary strength lies in its intuitive web interface for immediate data insights and its strong integration with MATLAB for advanced analytical tasks. It also supports popular hardware platforms like Arduino, ESP8266, ESP32, and Raspberry Pi through dedicated libraries. However, its SDK offerings are more focused on data interaction and less on broad application development compared to more comprehensive IoT platforms. This makes ThingSpeak an excellent tool for educational purposes, rapid prototyping of data-centric IoT projects, and applications requiring strong analytical capabilities, earning it a score of 7 out of 10 for its specialized and accessible developer resources.
- **ThingWorx** provides essential SDKs in Java, .NET, and C, crucial for connecting industrial assets and integrating with enterprise systems. Its comprehensive REST APIs and powerful visual development environment (ThingWorx Composer) significantly accelerate the development of industrial IoT applications and digital twins. While specialized for industrial contexts, its tools are robust and effective, earning a score of 8.

#### 5.1.8. Compliance and Standards

In the rapidly expanding and increasingly critical domain of the IoT, adherence to compliance frameworks and industry standards is not only a best practice but a fundamental requirement. These guidelines are essential for ensuring the Confidentiality, Integrity, and Availability (CIA) of IoT data and systems, alongside crucial aspects like non-repudiation. Given the widespread nature of IoT devices and their integration into sensitive sectors such as healthcare, critical infrastructure, and smart homes, robust cybersecurity practices are paramount. Standards and compliance frameworks offer structured, universally recognized guidelines that help mitigate cybersecurity risks, foster device interoperability, ensure secure communication, and protect sensitive data. For businesses, compliance reduces legal and financial risks, builds customer trust, and facilitates market access, making it a pivotal criterion for platform selection.

Table 8 provides a comparative overview of the compliance certifications and adherence to industry standards across the evaluated IoT Cloud Platforms.

Discussion per Platform:

- **Arduino IoT Cloud** is ISO/IEC 27001 certified [210] for information security, inheriting AWS’s broader compliance standards for data integrity, redundancy, and infrastructure security. While it does not yet offer certifications like SOC2 or FedRAMP, its security posture is robust and suitable for most use cases in healthcare, education, and industry. Considering these factors, we assign Arduino IoT Cloud a security score of 7 out of 10.
- **AWS IoT Core** benefits from Amazon Web Services’ extensive and continually updated list of compliance certifications. This includes a broad range of ISO certifications, industry-specific standards like Health Insurance Portability and Accountability Act (HIPAA) and Payment Card Industry Data Security Standard (PCI DSS), and government accreditations such as Federal Risk and Authorization Management Program (FedRAMP). This comprehensive adherence to global and regional standards provides customers with significant assurance regarding data security, privacy, and regulatory compliance for diverse IoT applications, earning it a score of 9.
- **Blynk**, primarily designed for smaller-scale projects and individual developers, currently has limited official compliance certifications beyond indicating that SOC2 compliance is “in process.” While it implements general security practices, the absence of a broad range of well-established certifications can be a barrier for enterprise deployments requiring stringent regulatory adherence. This limited formalized compliance results in a score of 4.
- **Bosch IoT Suite** demonstrates a strong commitment to compliance with key certifications like ISO 27001 (information security management), ISO 9001 (quality management), ISO/IEC 20000-1 (IT service management), and TISAX (information security in the automotive industry). These certifications highlight its suitability for industrial and enterprise-grade IoT applications, particularly within European regulatory environments. It receives a score of 7.
- **IoT on Google Cloud Platform**, which previously hosted IoT Core, is renowned for its robust compliance posture. It holds numerous global certifications, including a wide array of ISO standards, SOC reports, PCI DSS, HIPAA, FedRAMP, and FIPS 140-2, among others. While Google Cloud IoT Core itself has been deprecated, the underlying cloud infrastructure maintains these high standards, ensuring that data processed through its services would benefit from this compliance, earning it a score of 9.
- **Microsoft Azure IoT Hub** leverages Azure’s industry-leading compliance portfolio, which includes a comprehensive suite of ISO certifications, SOC reports, PCI DSS, HIPAA, FedRAMP, FIPS 140-2, and regional/national specific standards globally. This extensive and continuously updated list provides customers with strong assurance for even the most stringent regulatory environments, making it a top performer in this category, earning a score of 9.
- **Oracle IoT Cloud Service** benefits from Oracle’s enterprise-grade compliance framework, covering a wide range of certifications including multiple ISO standards, SOC reports, PCI DSS, FedRAMP, and NIST. This commitment to global and industry-specific compliance ensures that the platform meets the stringent requirements of large enterprises and regulated sectors, securing a score of 9.
- **Particle IoT Platform** has attained important certifications like ISO 27001 (information security management), ISO 27017 (cloud services security), ISO 27018 (personally identifiable information protection), and SOC 2. These certifications indicate a solid commitment to security and data handling best practices, making it suitable for professional applications. It receives a score of 7.
- **Siemens Insights Hub** focuses heavily on compliance relevant to industrial environments. Beyond ISO 27001, it adheres to IEC 62443-4-1, a crucial standard for industrial control system security. While its list of general cloud certifications might be less extensive than the hyper-scale providers, its targeted industrial compliance is very strong, making it highly suitable for its niche. This specialized focus earns it a score of 7.
- **ThingsBoard** is an open-source IoT platform that provides capabilities for device management, data collection, processing, and visualization. It emphasizes robust security measures, including role-based access control and diverse device authentication options such as Access Tokens, Basic MQTT Credentials, and X.509 Certificates. The platform also supports secure communication protocols like MQTT over SSL/TLS, crucial for protecting data in transit. ThingsBoard’s commitment to data protection is further highlighted by its adherence to GDPR principles, ensuring user data privacy. This makes ThingsBoard a secure and versatile choice for building various IoT solutions, earning it a score of 8 out of 10 for its comprehensive security framework and compliance considerations.
- **ThingSpeak** is an IoT analytics platform offered by MathWorks that allows users to collect, visualize, and analyze live data streams from sensors. It primarily focuses on ease of use for data aggregation and basic analytics, especially for MATLAB users. For security, ThingSpeak utilizes secure communication (SSL/TLS) for data transmission, relying on trusted certificates like DigiCert Global Root CA to ensure encrypted connections. It also employs API keys to control access to channels for both reading and writing data, providing a fundamental level of security for user-generated content. This makes ThingSpeak suitable for rapid prototyping and educational purposes, earning it a score of 6 out of 10 for its essential security features and ease of data handling.
- **ThingWorx** adheres to important certifications such as ISO 27001:2013 (information security management) and SOC 2. These demonstrate a commitment to foundational security and data management practices. However, compared to the broader and more diverse compliance portfolios of the major cloud providers that cover a multitude of industry-specific and regional regulations, ThingWorx’s listed compliance is somewhat less comprehensive, leading to a score of 6.

#### 5.1.9. OTA Update Capabilities

OTA updates refer to the wireless transmission of software updates, firmware patches, or configuration changes directly to IoT devices deployed in the field. This capability is paramount for the long-term viability, security, and functionality of any IoT ecosystem. From a cybersecurity perspective, OTA updates enable timely responses to emerging security threats, ensuring devices remain protected from known vulnerabilities and minimizing the risk of unauthorized access or exploitation [222]. Beyond security, OTA updates are crucial for deploying new features, fixing bugs, optimizing device performance, and adapting to changing requirements without the need for physical access, thereby reducing maintenance costs and extending the effective lifespan of devices. Robust OTA mechanisms typically include features for secure delivery, version control, fleet management, and rollback capabilities in case of faulty updates.

Table 9 compares the OTA update capabilities offered by the evaluated IoT Cloud Platforms, detailing their features and supported device platforms.

Discussion per Platform:

- **Arduino IoT Cloud** supports OTA firmware updates [223] for all compatible boards, including verified ESP32/ESP8266 devices, as well as for several Arduino MKR/Nano/Porenta/Nicla devices [224]. The update process is managed via the Web interface or the Arduino CLI using Arduino Sketches [225]. Enterprise users gain access to fleet-wide firmware rollout features and secure OTA updates for devices like the high-performance board Portenta X8 [226], which runs Linux OS (Yocto). This makes Arduino IoT Cloud one of the most flexible OTA platforms in its class, earning it a score of 9 out of 10 for its comprehensive and accessible OTA capabilities.
- **AWS IoT Core** provides highly capable and scalable OTA update functionalities through AWS IoT Device Management, specifically leveraging AWS IoT Jobs and integration with the FreeRTOS OTA agent. This allows for secure, targeted, and highly reliable firmware and software updates across large fleets of devices, with features like staggered rollouts and progress monitoring. Its deep integration with a widely used RTOS makes it very effective, earning a score of 9.
- **Blynk**. Blynk offers the “Blynk.Air” service, a convenient solution for remote firmware updates, particularly for Wi-Fi-connected devices. It supports common microcontrollers like ESP32 and Arduino, providing essential features like device-side checks and basic rollback. While highly effective and user-friendly for its target audience of hobbyists and small-scale deployments, its capabilities might be less comprehensive for large-scale, enterprise-grade deployments requiring advanced fleet management across diverse connectivity types. This leads to a score of 7.
- **Bosch IoT Suite** provides robust OTA capabilities through Bosch IoT Rollouts. This service offers comprehensive software update management, including secure update delivery, campaign management for staged rollouts, and essential rollback features to mitigate risks. Its design caters specifically to the demands of industrial IoT, ensuring reliable and secure updates for its supported devices, earning it a score of 8.
- **IoT on Google Cloud Platform**. As of August 2023, Google Cloud IoT Core has been deprecated. Consequently, there is no longer a dedicated, managed OTA update service provided directly within what was formerly IoT Core. While users might implement custom OTA solutions using other Google Cloud services (e.g., Cloud Storage for firmware and Cloud Functions for orchestration), the platform itself no longer offers this core functionality directly. This deprecation leads to a very low score of 3 out of 10.
- **Microsoft Azure IoT Hub** excels in OTA update capabilities with its device update for IoT Hub service. This robust solution enables highly scalable deployment of firmware and software updates to IoT devices, supporting various operating systems like Azure RTOS, Linux, and Eclipse ThreadX. It offers comprehensive management, monitoring, and crucial rollback capabilities, making it a leading choice for sophisticated device update strategies. This comprehensive feature set merits a score of 9.
- **Oracle IoT Cloud Service** (now often referred to under the umbrella of Oracle IoT Intelligent Applications) includes features for device administration and management, which inherently involve aspects of OTA updates. The platform is designed to facilitate secure firmware updates, offering capabilities for deployment, monitoring of update status, and basic version control. While comprehensive, explicit documentation on advanced features like granular rollout campaigns, extensive rollback capabilities, or detailed delta updates might require deeper investigation into their specific application services (e.g., Asset Monitoring and Production Monitoring). Devices typically connect via Oracle IoT Device SDKs, supporting languages like Java and C Posix, which would be instrumental in implementing the client-side logic for fetching and applying updates. The focus is on integrating IoT data and device management within the broader Oracle Cloud ecosystem for enterprise applications. This positions Oracle IoT Cloud Service as a solid option for organizations already invested in the Oracle stack, earning it a score of 7 out of 10 for its integrated OTA capabilities within an enterprise context.
- **Particle IoT Platform** offers strong and seamlessly integrated OTA update capabilities, particularly for its own hardware and Device OS. This tight integration ensures secure, reliable, and straightforward firmware updates directly from the cloud to Particle microcontrollers and System-on-Modules (SoMs). Its focus on a cohesive hardware-software experience makes OTA updates highly effective within its ecosystem, earning it a score of 8.
- **Siemens Insights Hub** provides specialized OTA update tools primarily designed for Siemens’ industrial devices and MindConnect gateways. These capabilities are crucial for maintaining and updating industrial control systems and connected machinery in Operational Technology (OT) environments. While robust for its industrial niche, its broader applicability for diverse non-Siemens devices might be more limited compared to general-purpose Cloud Platforms. This specialized strength earns it a score of 7.
- **ThingsBoard** offers a robust and comprehensive solution for OTA updates. Its key features include the ability to upload and manage firmware and software packages directly within the platform. Users can assign these packages to entire device profiles, enabling fleet-wide rollouts, or to individual devices for targeted updates. The platform provides detailed tracking of the update status, offering visibility into the progress and success of each deployment. ThingsBoard also incorporates basic queuing mechanisms to manage large-scale updates efficiently, preventing system overload. While it offers a versatile API for device integration, specific examples and guides, particularly for ESP32, highlight its practicality for common IoT development. Its open-source nature and strong community support further enhance its appeal for flexible and scalable IoT solutions. This makes ThingsBoard a highly capable and feature-rich platform for OTA updates, earning it a score of 8 out of 10.
- **ThingSpeak**, primarily recognized as an IoT analytics platform, offers more rudimentary OTA update capabilities compared to dedicated device management platforms. While it allows for data aggregation and visualization, its role in OTA updates is largely indirect. Devices typically need custom firmware or software that can communicate with ThingSpeak channels to retrieve update information, such as a URL to a new firmware file. This means the heavy lifting of managing the update process (downloading, verifying, and flashing) falls to the device-side code. It integrates well with popular microcontrollers like ESP8266, ESP32, and Arduino through its libraries, but it lacks the centralized campaign management, robust rollback features, and detailed status tracking found in more comprehensive IoT platforms. It is suitable for simpler OTA scenarios or when integrated with external update management tools, earning it a score of 5 out of 10 for its limited, yet functional, OTA support.
- **ThingWorx**, a leading IIoT platform by PTC, provides robust and comprehensive OTA update capabilities as a core part of its device management features. It enables secure deployment of firmware and software updates to connected devices at scale, which is crucial for industrial environments requiring high reliability and security. The platform offers capabilities for monitoring the status of updates across entire fleets, providing visibility into the progress and success rates of deployments. While it includes functionality for managing updates and deployment, documented specifics on sophisticated rollback mechanisms might require deeper exploration within its extensive documentation. ThingWorx’s strength lies in its deep integration with industrial protocols and its focus on enterprise-grade device management, making it highly suitable for complex, large-scale industrial deployments. This makes ThingWorx a powerful platform for managing device updates in demanding industrial settings, earning it a score of 9 out of 10.

### 5.2. Comparative Score Table

The following table presents a comparative score of various platforms across a range of critical features. Each platform received a score of 1 to 10, reflecting our assessment of its capabilities. This scoring is based on a comprehensive evaluation that incorporates expert judgment and comparative benchmarking against industry best practices.

Scores in Table 10 were ultimately assigned through a rigorous process of expert judgment, synthesizing information from all available sources. This process involved a multi-faceted comparison: each platform’s feature implementation was benchmarked against its competitors and against a defined ideal or industry best practice. For example, in evaluating a platform’s security, we considered not only the presence of features like MFA but also the ease of their implementation, their integration capabilities with other services, and applicable compliance certifications. The arithmetic mean, ranging from 5.33 to 8.89 as the final unweighted score, offers a clear indication of the relative strengths and weaknesses of each platform, across all evaluated features.

## 6. The Decision-Making Tool for Evaluation of IoT Cloud Platforms

While the comprehensive feature-driven evaluation presented in Section 5 offers invaluable insights into the capabilities of the leading IoT Cloud Platforms, the dynamic nature of project requirements and the inherent subjectivity of prioritization present another challenge. Recognizing that the “best” platform is ultimately context-dependent, this section introduces a novel, web-based tool for evaluating IoT Cloud Platforms, called the “IoT Cloud Platforms Selector”. This interactive utility bridges the gap between our static, expert-derived scores and the user’s specific needs, empowering them to dynamically weigh the importance of each feature. By translating individual priorities into quantifiable recommendations, the tool transforms a complex multi-criteria decision into a transparent and personalized selection process, offering a practical complement to our academic evaluation.

### 6.1. Tool Rationale and Design

The development of the “IoT Cloud Platforms Selector” stems from a fundamental recognition of two critical factors inherent to the IoT cloud landscape: (a) its highly dynamic environment and (b) the inherent diversity of user-specific needs and priorities. While our comprehensive feature-driven evaluation (as presented in Section 5) provides a robust, expert-derived snapshot of the leading platforms at a specific point in time, it represents a static assessment. The rapid pace of innovation in cloud computing means that platform capabilities evolve constantly, and, more importantly, the “best” platform for one organization may not be the “best” for another due to varying strategic objectives, existing infrastructure, budget constraints, or specific industry compliance requirements.

A static score, no matter how meticulously derived, cannot universally accommodate these multifaceted and fluid demands. For instance, a small startup prioritizing rapid prototyping and cost-effectiveness might value “Developer Tools & SDK Support” and “Pricing Models & Cost-effectiveness” far more highly than “Compliance & Standards” or “Edge Computing Support”, whereas a large enterprise in a regulated industry would likely place paramount importance on “Security” and “Compliance & Standards”. Existing comparative studies often present their findings as fixed assessments, leaving the burden of interpreting and applying these generalized insights to a user’s unique context entirely on the user.

To overcome these limitations, the core rationale behind our decision-making tool is to introduce user-defined customization and dynamic weighting. The fundamental concept is to transform our expert-assigned platform scores into a personalized recommendation by incorporating the user’s specific priorities. This is achieved through a weighted scoring model, where the user assigns each of the nine evaluation features a weight. These weights reflect the relative importance of each feature to the user’s particular IoT project or organizational strategy.

### 6.2. Algorithm for Ranking IoT Cloud Platforms

The core of our decision-making tool relies on a weighted scoring algorithm (detailed in Algorithm 1) to rank IoT Cloud Platforms based on user-defined priorities. The user provides these weights via intuitive slider controls (ranging from 1 to 10) for each feature on the tool’s interface. The algorithm begins by calculating a ‘‘Final Score’’ for each platform by multiplying each feature score (derived from our evaluation in Section 5) with the corresponding user-assigned weight, summing these weighted scores, and normalizing the result by the total sum of weights. This ensures that the platforms are ranked based on a score that accurately reflects the user’s feature preferences. In cases of ties, a multi-stage tie-breaking mechanism is applied, prioritizing features based on user-defined weight assignment, and finally sorting alphabetically by platform name.

Algorithm Mechanics:

Let $M\in N^{*}$ be the number of platforms being considered and $N\in N^{*}$ be the total number of evaluated features. For each platform *i*, its total weighted arithmetic mean, as final score $fs_{i}$, is calculated as the sum of its static scores from its feature vector $fv_{i}$ multiplied by the user-defined weights $\sum_{j=1}^{N}\left(fv_{i}\left[j\right]\times w_{j}\right)$, divided by the sum of these weights $\sum_{j=1}^{N}w_{j}$. This can be represented by Equation (1).

(1) $$fs_{i}=\frac{\sum_{j=1}^{N}\left(fv_{i}\left[j\right]\times w_{j}\right)}{\sum_{j=1}^{N}w_{j}}$$

where $N^{*}=N-\left\{0\right\}$.

This calculation is designed to ensure the ranking accurately reflects user feature preferences. In cases of identical final scores among platforms, a robust, multi-stage tie-breaking mechanism, outlined in Algorithm 1, is employed. This comprehensive approach guarantees a precise and personalized ranking system, offering users clear, tailored recommendations.
**Algorithm 1:** Platform-Ranking Algorithm
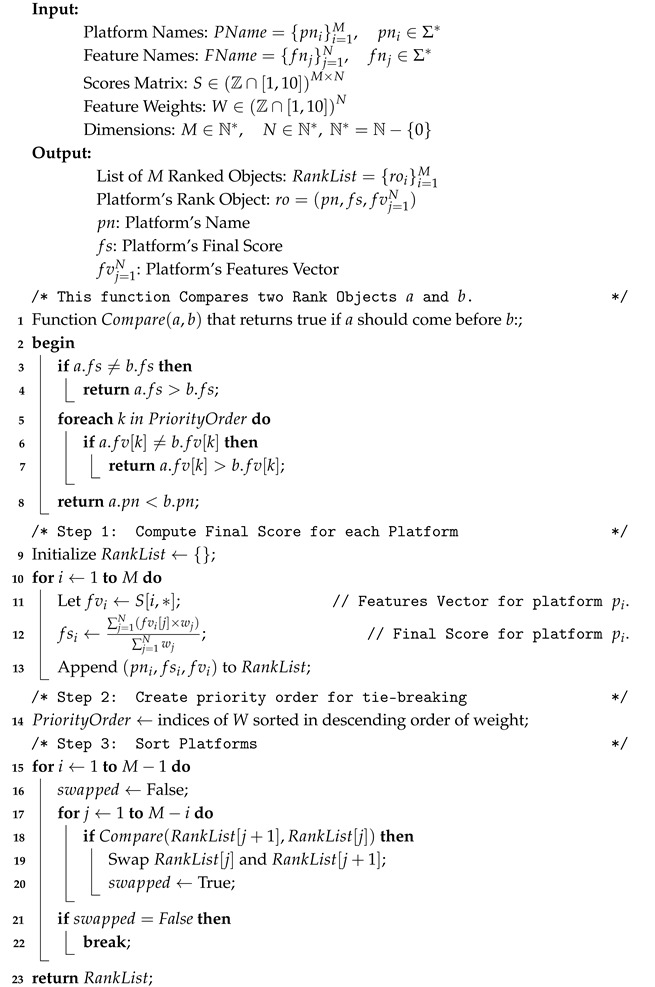


### 6.3. Technical Implementation

The “IoT Cloud Platforms Selector” was developed as a web-based application to maximize accessibility and ease of use, allowing users to run it on any device with a standard web browser without software installation. The tool is architected as a purely client-side application and does not have a server-side backend. All logic is executed directly within the user’s browser. The frontend is built with standard web technologies: HTML5 structures the content, CSS3 handles the responsive styling, and JavaScript powers all interactive functionalities. To create the dynamic bar and radar charts for data visualization, the open-source Chart.js (v4.4.9) library was used. The core logic for ranking the platforms is implemented directly in the client-side JavaScript. Table 10, which depicts the static platform scores (derived from our comprehensive evaluation in Section 5), is embedded within the application. When a user adjusts the feature weight sliders, the application instantly recalculates the platform rankings based on the weighted scoring algorithm detailed in Section 6.2 (Algorithm 1, Equation (1)). This client-side architecture ensures real-time feedback and a seamless user experience without server latency. For complete transparency and to encourage reuse and collaboration, the tool is open source, released under the GPL License. The full source code is available in the GitHub repository listed in the “Data Availability Statement” at the end of this manuscript.

### 6.4. User Interface and Functionality

The “IoT Cloud Platforms Selector”, which is developed as part of this study, offers an intuitive and interactive user interface designed to facilitate the comparison and selection of IoT cloud platforms based on user-specific preferences.

**Figure 1:**
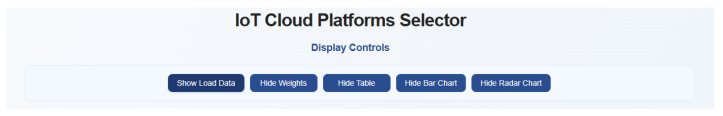
IoT Cloud Platforms Selector: Control Panels. These buttons controls the displayed panels on the application canvas.

The tool’s interface provides control panels with toggle buttons (Figure 1) to show or hide various components such as data tables, weight sliders, bar charts, and radar charts, allowing users to customize their view according to their preferences.

**Figure 2:**
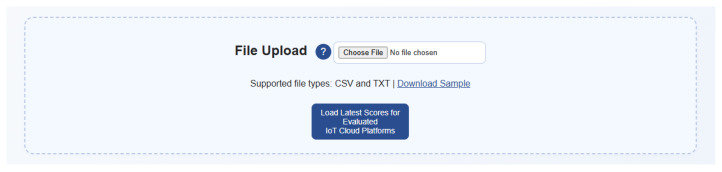
IoT Cloud Platforms Selector: Data Panel. Loads the latest scores derived from our comprehensive evaluation in Section 5 or loads custom data in csv format.

Users can upload custom datasets in CSV or TXT formats through the data panel (Figure 2) or, alternatively, load the default dataset that contains the evaluated platforms’ latest scores from our research team. This flexibility supports both standard and custom analyses.

**Figure 3:**
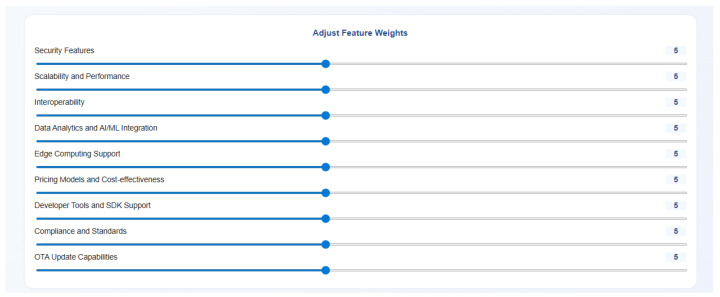
IoT Cloud Platforms Selector: User-defined Feature Weights. The user is able to assign personalized weights to each feature.

At the core of the UI are sliders that allow users to assign custom weights to each evaluation feature on a scale from 1 to 10, reflecting the relative importance of each criterion in their decision-making process (see Figure 3). These weights are then used to compute a weighted score for each IoT cloud platform by multiplying the feature scores (derived from our comprehensive evaluation detailed in Section 5) by the corresponding user-defined weights and aggregating the results. This approach enables personalized ranking aligned with individual priorities.

**Figure 4:**
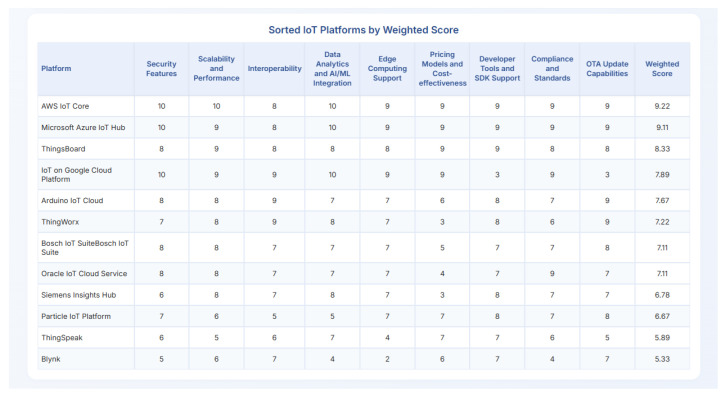
IoT Cloud Platforms Selector: A Panel with sorted IoT Cloud Platforms by Weighted Score.

**Figure 5:**
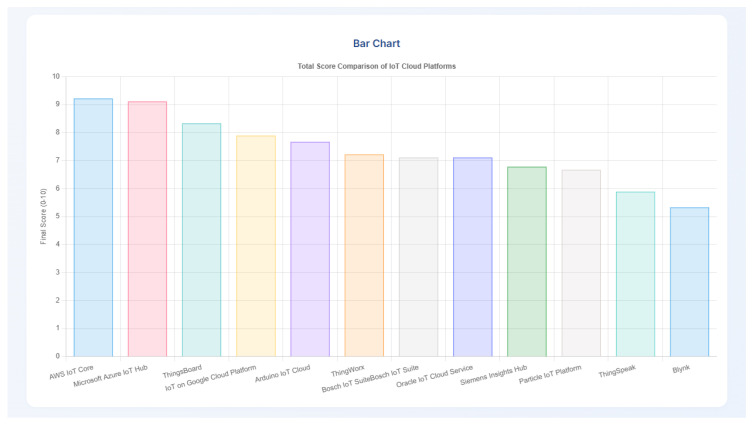
IoT Cloud Platforms Selector: A Bar Chart with Sorted IoT Cloud Platforms by Weighted Score.

**Figure 6:**
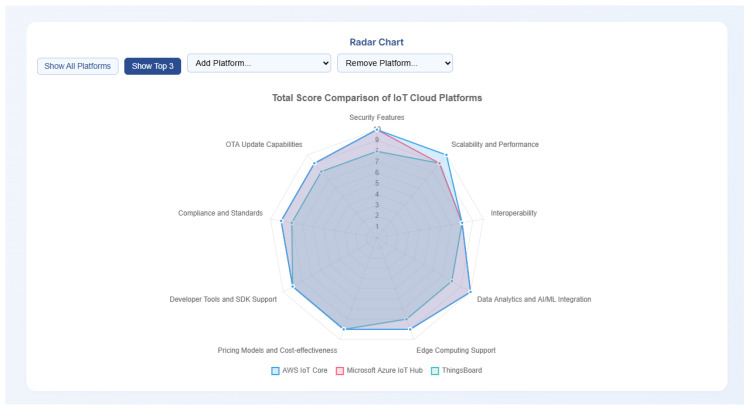
IoT Cloud Platforms Selector: A Radar Chart with Weighted Features of all IoT Cloud Platforms.

The computed results are displayed in multiple formats to aid decision-making:

- A sorted list of IoT platforms in descending order by their weighted scores (Figure 4), providing a clear textual ranking.
- A bar chart visualization (Figure 5) that graphically represents the platforms’ weighted scores for quick comparison.
- A radar chart (Figure 6) that illustrates the weighted feature profiles of all platforms, highlighting strengths and weaknesses across multiple dimensions.

Developed at the TelSiP Research Laboratory, this tool is released under the GPL License, ensuring accessibility and adaptability for the research community and practitioners alike. Its comprehensive functionality empowers users to perform nuanced evaluations tailored to their specific IoT deployment needs.

### 6.5. Benefits and Use Cases

The “IoT Cloud Platforms Selector” offers substantial benefits across various stakeholders and use cases, significantly simplifying the complex process of IoT platform selection.

One of the primary benefits is its enhanced decision-making capability. By allowing users to assign personalized weights to different features, the tool moves beyond a generic ranking and provides a truly tailored evaluation. This customization ensures that the selected platform aligns precisely with an organization’s specific priorities, whether those are security, scalability, cost-effectiveness, or a combination thereof. This nuanced approach helps mitigate the risk of choosing a suboptimal platform, which can lead to increased development costs, operational inefficiencies, or even project failure.

Furthermore, the tool promotes transparency and objectivity in the selection process. The quantitative scoring mechanism and the clear visual representations (bar charts, radar charts) make the rationale behind each platform’s ranking explicit. This reduces reliance on subjective opinions or anecdotal evidence, fostering a more data-driven and defensible decision. For organizations, this means a more rigorous procurement process and improved accountability.

The time and resource efficiency offered by the tool are also significant. Manually evaluating numerous IoT Cloud Platforms across multiple features is a time-consuming and labor-intensive task. The automation provided by the tool streamlines this process, allowing users to quickly assess and compare platforms, thereby accelerating the decision cycle and freeing up valuable resources for other critical tasks.

The flexibility and adaptability of the tool is demonstrated by its ability to upload custom datasets. This feature makes it highly valuable for researchers and practitioners who wish to incorporate their own evaluation data or analyze emerging platforms not included in the default dataset. This ensures that the tool remains relevant and useful in the rapidly evolving IoT landscape.

Several key use cases can leverage the benefits of this decision-making tool:

- **Enterprise IoT Solution Architects**: Architects designing large-scale IoT deployments can use the tool to identify platforms that best meet their organization’s specific requirements for security, compliance, integration with existing systems, and performance at scale.
- **Small and Medium-sized Enterprises (SMEs) and Startups**: For businesses with limited resources and expertise in IoT, the tool provides a structured and simplified approach to platform selection, helping them avoid costly mistakes and accelerate their time to market.
- **IoT Developers and Engineers**: Individual developers or teams can use the tool to compare platforms for specific projects, considering factors like developer tools, SDK support, and ease of use to optimize their development workflow.
- **Academic Researchers and Educators**: The tool serves as an excellent resource for research studies on the capabilities of IoT platforms and for educational purposes, allowing students to explore the impact of different feature priorities on platform selection.
- **Consulting Firms**: Consultants advising clients on IoT strategies can utilize the tool to provide data-backed recommendations tailored to their clients’ unique business needs and technical constraints.
- **Proof-of-Concept and Pilot Projects**: Before committing to a full-scale deployment, organizations can use the tool to narrow down options for PoC or pilot projects, quickly identifying suitable platforms for initial testing and validation.

In essence, the “IoT Cloud Platforms Selector” empowers users to navigate the intricate world of IoT platforms with confidence, leading to better-informed decisions, optimized resource allocation, and, ultimately, more successful IoT initiatives.

## 7. Discussion and Limitations

In this section, we synthesize the core insights derived from our comprehensive evaluation of IoT Cloud Platforms. We will delineate the prevailing trends, distinguish platform-specific strengths, and identify common deficiencies. Furthermore, we discuss the practical and research implications of these findings. It is imperative to also acknowledge the inherent limitations of our methodology and analytical scope as a thorough understanding of both the derived knowledge and its boundaries is critical for sound decision-making and the accurate direction of subsequent research.

### 7.1. Key Findings and Insights

Our comprehensive evaluation revealed several key trends across the assessed IoT Cloud Platforms. Generally, platforms demonstrate a strong focus on core connectivity and device management capabilities, indicating maturity in these fundamental areas. However, significant variation exists in more advanced features such as data analytics, edge computing support, and robust security implementations.

Several platforms consistently excel in specific domains. For example, platforms with a strong enterprise focus consistently scored higher in security and compliance features, likely due to the stringent requirements of their target market. Others demonstrated leadership in data analytics capabilities, offering sophisticated tools for real-time data processing and visualization.

In contrast, a common weakness observed across many platforms was the lack of seamless integration with legacy systems. This presents a significant challenge for organizations seeking to modernize their IoT infrastructure while preserving existing investments. Furthermore, documentation and ease of use varied considerably, creating a steeper learning curve for some platforms. A notable observation was that while many platforms offer a comprehensive feature set, practical implementation and user experience were not always prioritized, hindering their overall effectiveness. Understanding these nuances is vital when choosing a platform to support specific IoT initiatives.

### 7.2. Challenges in Evaluation

The evaluation of IoT Cloud Platforms presented several inherent challenges. The rapidly evolving nature of cloud technologies meant that platform features and capabilities were subject to frequent updates, sometimes occurring mid-evaluation. This required continuous monitoring and adaptation of our assessment criteria to ensure accuracy and relevance.

Another significant hurdle was the varying quality of the documentation provided by different vendors. Some platforms offered comprehensive and well-maintained documentation, while others lacked sufficient detail or were outdated, hindering our ability to fully understand and assess their capabilities.

Furthermore, thoroughly testing certain enterprise-grade features, such as advanced security protocols and high-availability configurations, proved difficult without significant investment in platform-specific infrastructure and resources. Access to fully functional trial environments was often limited, making it challenging to conduct realistic performance and scalability testing. Finally, objectively comparing features offered through different pricing tiers or subscription models required careful consideration and normalization to ensure a fair evaluation. Addressing these challenges required a flexible and iterative approach, coupled with a reliance on a combination of publicly available information, vendor-provided documentation, and expert judgment.

### 7.3. Limitations of the Study

Although this evaluation provides a comprehensive overview of the leading IoT Cloud Platforms, it is important to acknowledge certain limitations that frame the context of our findings.

- **Snapshot in a Dynamic Field:** The scores and analysis represent a snapshot in time. The IoT cloud landscape is highly volatile, with vendors constantly releasing updates, altering services, and adjusting pricing models. A prime example of this dynamism is the deprecation of Google Cloud IoT Core, which was announced in 2022 and took effect in August 2023. This event, while preceding our research period, fundamentally changed the competitive landscape. This dynamism means that while our framework is robust, the specific scores and vendor standings may lose accuracy over time.
- **Inherent Scoring Subjectivity:** While we employed a systematic methodology and a consensus-building protocol to mitigate bias, some degree of subjectivity in the scoring process is unavoidable. Expert judgment was used to assess qualitative aspects like user-friendliness and documentation quality, and different experts could arrive at different conclusions.
- **Non-Exhaustive Scope:** The study, while comprehensive, is not exhaustive. We evaluated twelve prominent platforms, but numerous other specialized or emerging platforms exist in the market. Similarly, the nine features were chosen for their critical importance, but other niche or specific functionalities were not included in the analysis.
- **Lack of Real-World Load Testing:** Our evaluation is primarily based on publicly available documentation, vendor materials, and hands-on testing within trial or free-tier environments. Extensive, real-world load testing and performance benchmarking under a variety of high-stress conditions were beyond the practical scope of this study. Therefore, actual performance may vary depending on the specific scale and workload of a deployment.

Recognizing these limitations is crucial to interpreting the results and making informed decisions.

### 7.4. Future Work

This evaluation serves as a foundational step, and there are several avenues for future research and development. A key priority is to establish a process for regularly updating the platform scores and the associated decision-making tool to reflect the rapidly evolving landscape of IoT cloud technologies. Expanding the scope of the evaluation to include a wider range of platforms and features would further enhance its comprehensiveness and value. Incorporating qualitative feedback from actual users—through surveys, interviews, or case studies—would provide valuable insights into real-world experiences and usability. Developing more sophisticated weighting algorithms, perhaps using machine learning techniques, could allow for a more nuanced and personalized assessment of platform suitability based on specific user requirements. Integrating cost optimization models into the decision-making tool would enable a more holistic evaluation, considering not only functional capabilities but also the total cost of ownership.

A more granular analysis of interoperability could also be undertaken. While our current evaluation focuses on the critical layer of communication protocol support, true interoperability in IoT involves multiple levels, including syntactic interoperability (e.g., standardized data formats like JSON or CBOR) and semantic interoperability (e.g., shared data models and ontologies like ETSI’s oneM2M or W3C’s Web of Things). Future studies could provide immense value by evaluating platforms on their support for these deeper layers, which are crucial for enabling meaningful data exchange between heterogeneous systems and preventing the creation of functional data silos.

Additionally, future work should explore the cross-correlations and inter-dependencies between the evaluated features. Our model treats each feature as an independent variable for the purpose of a clear scoring framework, but in practice, they are often interrelated. For example, enhancing Security might introduce performance overhead that affects Scalability and Performance or require premium services that impact Pricing and Cost-effectiveness. Similarly, a platform’s commitment to Interoperability and open standards could influence long-term cost by preventing vendor lock-in. Analyzing these trade-offs would require a more complex, multi-objective evaluation model and represents a significant avenue for future investigation, leading to an even more nuanced understanding of platform selection.

Finally, future work could focus on considering specific industry vertical requirements. Different sectors, such as healthcare, manufacturing, and retail, often have unique needs and priorities, and tailoring the evaluation criteria accordingly would provide more relevant and actionable insights.

## 8. Conclusions

This paper has presented a comprehensive, feature-driven evaluation of twelve leading IoT Cloud Platforms, meticulously analyzing them against nine critical features: Security, Scalability and Performance, Interoperability, Data Analytics and AI/ML Integration, Edge Computing Support, Pricing Models and Cost-effectiveness, Developer Tools and SDK Support, Compliance and Standards, and OTA Update Capabilities. The analysis revealed a dynamic and competitive landscape where major providers such as AWS IoT Core, Microsoft Azure IoT Hub, and Google’s IoT offerings demonstrate superior capabilities in scalability, security, and extensive AI/ML integration, making them suitable for large-scale enterprise deployments. In parallel, platforms like Arduino IoT Cloud, Blynk, and Particle offer highly accessible, developer-friendly environments that excel in rapid prototyping and serving niche applications.

The primary contribution of this research is the introduction of a novel, web-based tool for evaluating IoT Cloud Platforms, called the “IoT Cloud Platforms Selector”. This interactive utility bridges the gap between the provided static, expert-derived evaluation and the unique, context-dependent needs of users. By allowing users to assign personalized weights to each feature, the tool transforms a complex, multi-criteria decision into a transparent, personalized, and data-driven recommendation process. This addresses a significant gap in the literature by providing a practical instrument that moves beyond generalized comparisons to facilitate the selection of a customized platform.

Although core functionalities such as device connectivity and management have reached a level of maturity across most platforms, there is significant differentiation in advanced domains. The strength of a platform’s edge computing support, its seamless AI/ML integration, and its adherence to a broad range of compliance standards are key factors that distinguish the leading solutions. It is critical, however, to acknowledge the limitations of the study. The evaluation represents a snapshot in time in a rapidly evolving market, and the quantitative scores, while rigorously derived, contain an element of expert judgment.

Future work should focus on maintaining the relevance of this research by regularly updating the platform scores to reflect the continuous evolution of the IoT cloud landscape. Expanding the evaluation to include a wider range of platforms, incorporating qualitative user feedback, and tailoring criteria for specific industry verticals would further enhance the value of this work for the community.

In conclusion, this study provides a significant resource for the IoT community. This research aims to assist future researchers, developers, and organizations by providing a systematic framework to navigate the complex ecosystem of IoT Cloud Platforms. The presented framework is a combination of a detailed comparative analysis with a powerful and adaptable decision-making tool, enabling better informed decisions and more successful IoT initiatives.

## Figures and Tables

**Table 1:** Comparison of security features across IoT Cloud Platforms.

Platform	References	Cryptographic Protocols	Authentication Mechanisms	Score
Arduino IoT Cloud	[68,69,70,71]	TLS 1.2 (BearSSL)	X.509 certificates, Arduino IoT Cloud’s backend is hosted on AWS, ISO/IEC 27001 certified, 2FA.	8
AWS IoT Core	[72,73]	TLS 1.2, 1.3	X.509 certificates, AWS credentials, Amazon Cognito identities, federated identities, or custom authentication token.	10
Blynk	[74,75]	TLS 1.2, 1.3	OAuth	5
Bosch IoT Suite	[76]	TLS 1.2, 1.3	OAuth2, X.509 Certificates, API Keys.	8
IoT on Google Cloud Platform	[77,78]	TLS 1.2, 1.3	Oauth, X.509 Certificates, API keys, Service account keys, Google IAM, Application Default Credentials	10
Microsoft Azure IoT Hub	[79,80]	TLS 1.2, TLS 1.3, (TLS 1.0, TLS 1.1 planned for deprecation)	X.509 certificates, Trusted Platform Module (TPM), Symmetric key, Shared symmetric key.	10
Oracle IoT Cloud Service	[81]	TLS 1.2, TLS 1.3	REST resources over HTTP, OAuth.	8
Particle IoT Platform	[82,83,84]	DTLS over UDP (datagram TLS), AES over TCP	Postman, OAuth, Access Token, Username/Password.	7
Siemens Insights Hub	[85,86]	TLS 1.0, TLS 1.1, TLS 1.2	OAuth2, X.509 Certificates.	6
ThingsBoard	[87,88,89]	TLS (for MQTT over SSL/TLS, HTTPS)	Basic authentication (username/password/clientId), X.509 certificates, JWT tokens, Two-Factor Authentication (email, SMS, authenticator app, backup code).	8
ThingSpeak	[90,91]	TLS 1.2 (for HTTPS and MQTT)	API Keys (Write API Key, Read API Key, User API Key)	6
ThingWorx	[92,93]	SSL, TLS, AES	Username/Password, API Keys, Single Sign-On (SSO).	7

**Table 2:** Comparison of scalability and performance across IoT Cloud Platforms.

Platform	References	Scalability	Performance	Score
Arduino IoT Cloud	[69,95,96]	MODERATE	Not disclosed (Reliant on AWS)	8
AWS IoT Core	[97]	HIGH	Default value: 500 messages per second. In select AWS Regions*: 100 messages per second.	10
Blynk	[98]	LOW	Max number of requests per device: 50 requests per second.	6
Bosch IoT Suite	[99]	MODERATE	Standard plan supports up to 1,000,000,000 messages per tenant per month.	8
IoT on Google Cloud Platform	[100]	HIGH	100 messages per second, per device.	9
Microsoft Azure IoT Hub	[101]	HIGH	5000 send operations per minute per unit.	9
Oracle IoT Cloud Service	[102]	HIGH	Performance details not disclosed (Uses Oracle Cloud Infrastructure Platform).	8
Particle IoT Platform	[103]	LOW	1 event per second limitation.	6
Siemens Insights Hub	[104]	MODERATE	Designed to handle thousands of messages per second in enterprise environments, particularly for applications like manufacturing and smart infrastructure.	8
ThingsBoard	[88,105]	HIGH	Enterprise plans support up to 20,000 messages per second per tenant. Open-source deployments depend on infrastructure.	9
ThingSpeak	[106,107]	LOW	Free plan: 1 message per 15 s. Standard License: 1 message per second.	5
ThingWorx	[108]	MODERATE	Designed to support high message rates for industrial applications, potentially handling thousands of messages per second in enterprise configurations.	8

**Table 3:** Comparison of interoperability across IoT Cloud Platforms.

Platform	References	Supported Protocols	Score
Arduino IoT Cloud	[110,111,112,113,114,115,116]	MQTT, HTTPS, REST API, CoAP, AMQP, XMPP, DDS	9
AWS IoT Core	[117]	MQTT, HTTPS, WebSockets	8
Blynk	[118,119]	Blynk Protocol, HTTPS, MQTT	7
Bosch IoT Suite	[120]	MQTT, HTTPS, AMQP	7
IoT on Google Cloud Platform	[58]	MQTT, HTTPS, REST/gRPC APIs, Google Cloud Pub/Sub, and other protocols (e.g., LoRaWAN, CoAP) via integration with third-party systems.	9
Microsoft Azure IoT Hub	[121]	MQTT, HTTPS, AMQP, WebSockets	8
Oracle IoT Cloud Service	[122]	MQTT, HTTPS	7
Particle IoT Platform	[123]	MQTT, CoAP	5
Siemens Insights Hub	[124]	MQTT, HTTPS	7
ThingsBoard	[125,126]	MQTT, MQTT Sparkplug, HTTP, CoAP, LwM2M, SNMP, REST API	8
ThingSpeak	[90,127]	MQTT, HTTP	6
ThingWorx	[128]	AlwaysOn (HTTPS, WebSocket), MQTT, OPC, ODBC, SNMP, REST APIs, and other protocols available in PTC Store	9

**Table 4:** Comparison of data analytics and AI/ML Integration across IoT Cloud Platforms.

Platform	References	Analytics	AI/ML Integration	Score
Arduino IoT Cloud	[131,132,133,134,135,136,137]	Real-time data monitoring, historical data viewing/export, customizable dashboards.	ML tools Addon (Edge Impulse), TensorFlow Lite/Google LiteRT, integration with external ML systems (AWS SageMaker, Google Colab, Azure ML).	7
AWS IoT Core	[55]	Integrates with AWS Analytics tools (AWS IoT Analytics, QuickSight) for data insights and anomaly detection.	Seamlessly integrates with AWS SageMaker and AI services for predictive analytics and ML models.	10
Blynk	[138,139]	Basic analytics and visualization tools for simple IoT applications, with focus on app-based data visualization.	Limited AI/ML integration; users can export data for use in external AI/ML platforms if needed.	4
Bosch IoT Suite	[140,141]	Offers Bosch IoT Analytics for real-time monitoring, data analysis, and anomaly detection.	Supports integration with Bosch ML solutions and external AI platforms for advanced analytics.	7
IoT on Google Cloud Platform	[142,143,144]	Supports BigQuery and Dataflow for scalable analytics and real-time data processing.	Integrates with Google AI Platform for advanced ML models and predictive analytics applications.	10
Microsoft Azure IoT Hub	[59]	Uses Azure Stream Analytics and Power BI for real-time data processing and visualization.	Supports Azure Machine Learning and Cognitive Services for predictive maintenance and anomaly detection.	10
Oracle IoT Cloud Service	[60]	Provides Oracle Analytics for IoT, with machine-learning-based anomaly detection and predictive analytics.	Uses Oracle Machine Learning services, allowing predictive insights and anomaly detection.	7
Particle IoT Platform	[145,146]	Includes basic analytics and data visualization tools for real-time monitoring and performance tracking.	Minimal AI/ML support; users can export data to integrate with external ML platforms.	5
Siemens Insights Hub	[147,148]	Provides 1. Processing (e.g., Visual Flow Creator); 2. Analytics (e.g., Insights Hub Quality Prediction); 3. Visualization (e.g., Insights Hub Business Intelligence, Insights Hub Monitor).	Integrates with AI/ML tools, such as Siemens AI and external ML platforms, for predictive maintenance insights.	8
ThingsBoard	[149,150]	Offers advanced analytics including real-time monitoring, historical data analysis, anomaly detection, and forecasting through its Trendz Analytics module. Provides customizable dashboards for data visualization.	Integrates with AI/ML models for predictive maintenance and forecasting. Trendz Analytics supports custom Python models and configuration with various LLMs (e.g., OpenAI, Amazon Bedrock, Google Gemini).	8
ThingSpeak	[64,151]	Provides real-time data aggregation, visualization, and analysis with built-in MATLAB analytics. Supports online processing, custom charting, and event scheduling.	Leverages MATLAB’s capabilities for building predictive algorithms, machine learning models, and deploying analytics in the cloud or at the edge.	7
ThingWorx	[152,153]	Advanced analytics via ThingWorx Analytics for real-time insights and anomaly detection.	Native support for AI/ML models; integrates with external ML platforms to enhance predictive capabilities.	8

**Table 5:** Comparison of edge computing support across IoT Cloud Platforms.

Platform	References	Products	Score
Arduino IoT Cloud	[155,156,157,158]	Arduino PRO-Edge IoT technology	7
AWS IoT Core	[159]	AWS IoT Greengrass, which enables AWS Lambda functions locally.	9
Blynk	[160,161,162]	Primarily cloud based; edge processing depends heavily on device-side logic and firmware (e.g., Blynk.Edgent for OTA). No officially supported local server for current platform.	2
Bosch IoT Suite	[163]	Bosch IoT Edge Agent and Edge Services.	7
IoT on Google Cloud Platform	[164]	Google Cloud IoT Edge.	9
Microsoft Azure IoT Hub	[165]	Azure IoT Edge.	9
Oracle IoT Cloud Service	[166]	Oracle Roving Edge Infrastructure.	7
Particle IoT Platform	[145,146]	Edge ML.	7
Siemens Insights Hub	[167,168]	MindConnect and Industrial Edge.	7
ThingsBoard	[169,170]	ThingsBoard Edge	8
ThingSpeak	[171]	Limited, primarily cloud based with local analysis capabilities via MATLAB	4
ThingWorx	[172]	ThingWorx Edge MicroServer (EMS).	7

**Table 6:** Comparison of pricing models and cost-effectiveness across IoT Cloud Platforms.

Platform	References	Pricing Models	Cost-Effectiveness	Score
Arduino IoT Cloud	[53,173]	Tiered pricing model. Free Plan for 2 devices, 100 MB data, 10 days data retention. Maker Plan (USD 6.99/month, 1 user, 25 devices, 1 GB data, 365 days data retention), Scalable Enterprise Plan available via AWS Marketplace (USD 5000/year, 500 devices), Prototype Plan (USD 240/year, 20 devices).	Accessible to SMEs and hobbyists. The Free and Maker plans offer a good starting point, while the Enterprise plan scales for larger deployments.	6
AWS IoT Core	[174]	Pay-as-you-go model. Free Tier: 500,000 messages/month for the first 12 months. After free tier: USD 1.00 per million messages (up to 1 billion), with volume discounts for higher usage. Additional costs for connectivity (USD 0.08 per million minutes connected), Device Shadow, Rules Engine, and other integrated services (e.g., S3 for storage, Lambda for compute).	Highly cost-effective for scalable growth from small to very large deployments, with granular control over costs. Can become complex with many interconnected services.	9
Blynk	[175]	Tiered pricing model. Free Tier: 2 devices, 5 users, 3 device templates, 5 datastreams per template, 1 week historical data. Plus Plan (USD 6.99/month): 10–20 devices/users, 10 device templates, 20 datastreams per template, 1 month historical data. Pro Plan (USD 49/month): 40–500 devices/users, 50 device templates, 200 datastreams per template, 6 months historical data. Business plans (e.g., USD 599/month, up to 10,000 devices/users, custom branding, private server).	Very cost-effective for small-scale projects, rapid prototyping, and personal/small business use due to simplified, tiered plans. Scales well for larger needs with clear pricing.	6
Bosch IoT Suite	[176,177]	Subscription-based, factoring in number of devices, data traffic, and specific service consumption. Specific pricing details are generally not publicly listed and require direct contact with Bosch.	Cost-effective for industrial and enterprise applications, especially those integrating with Bosch’s ecosystem. Offers predictable costs for managed services but can have a higher entry barrier for smaller projects due to bespoke pricing.	5
IoT on Google Cloud Platform	[178]	Pay-per-use for data ingest and egress, with additional costs for integrated services (e.g., BigQuery, AI Platform, Cloud Storage, Pub/Sub, Cloud Monitoring, Cloud Logging). Google Cloud offers a USD 300 free credit for new users across all services, including IoT. Data ingestion for monitoring is priced at USD 0.2580/MiB for the first 150–100,000 MiB.	Generally highly cost-effective for scalable deployments, offering flexibility to grow with a pay-as-you-go model. Requires careful management of integrated service costs.	9
Microsoft Azure IoT Hub	[179]	Tiered (Basic and Standard) based on number of messages/month and data transfer. Free Tier: 8000 messages/day. Basic (B1): USD 10/month per IoT Hub unit (400,000 messages/day). Standard (S1): USD 25/month per IoT Hub unit (400,000 messages/day, plus advanced features like cloud-to-device messaging, device twins). Higher tiers (B2, S2, B3, S3) are available for increased message quotas and features, up to USD 2500/month per S3 unit. Pay-as-you-go for linked services like Device Provisioning Service and Device Update.	Highly cost-effective for a wide range of deployment sizes, with predictable tiered pricing and extensive integration. Free and Basic tiers make it accessible for small projects, while Standard tiers support enterprise needs.	9
Oracle IoT Cloud Service	[60]	Device-based pricing per month with a fixed number of messages included; additional services metered. Specific pricing details are not readily public and often require direct consultation. Oracle’s general cloud pricing is based on a flexible pay-as-you-go or Universal Credits model.	Effective for enterprises already within Oracle’s ecosystem. Can be less flexible or costly for projects outside this integration, and pricing transparency may be lower compared to other hyperscalers.	4
Particle IoT Platform	[180]	Tiered subscription plans based on number of devices, data operations, and features. Offers a Free Tier. Specific current pricing for all tiers is often custom-quoted, but historically included plans like a “Developer” plan for small scale and “Enterprise” for large deployments. It provides integrated hardware and cloud services.	Highly cost-effective for startups, developers, and small-to-medium deployments focused on hardware–cloud integration due to its integrated nature and ease of use.	7
Siemens Insights Hub	[181]	Subscription-based, factoring in number of devices, data model complexity, and usage of specific applications/analyzers. Pricing is typically custom and enterprise-focused, not publicly disclosed. It is presented as an “Open IoT Operating System” with various modules.	Most economical for manufacturing and industrial enterprises heavily invested in Siemens’ ecosystem. High entry cost and tailored pricing for others, reflecting its focus on large-scale industrial applications.	3
ThingsBoard	[105,182,183]	Hybrid model: Open-source Community Edition (free for self-hosting). Professional Edition (PE) with tiered, device-based subscription for both Cloud and Self-Managed options. Cloud plans range from Maker (USD 10/month, 30 devices) to Business (USD 749/month. 1,000 devices). Enterprise plans are custom. PE Self-Managed licenses are perpetual or subscription-based, with costs varying significantly based on scale and additional features like Trendz Analytics.	Highly cost-effective for developers and small projects with the free Community Edition. Cloud and PE plans offer good scalability with predictable tiered pricing, balancing features with cost. Ideal for those seeking open-source flexibility combined with commercial support/features.	9
ThingSpeak	[107,184,185]	Tiered pricing with a focus on messages per year and channels. Free service for small non-commercial projects (<3 million messages/year, 4 channels, 15 s update interval). Home License (e.g., USD 95/year per unit for 33 million messages/year, 10 channels). Standard License for commercial use (e.g., USD 766.00/year per unit for 33 million messages/year, 250 channels, 1 s update).	Very cost-effective for hobbyists, educational purposes, and small-scale non-commercial projects due to its free tier. Scalable for larger commercial or home projects with clear, unit-based pricing, especially beneficial for users leveraging MATLAB analytics.	7
ThingWorx	[186]	Subscription based. Pricing available upon request; often tailored to enterprise needs. TrustRadius indicates it can be compared to AWS IoT Core and Azure IoT Hub in terms of cost. Focuses on industrial IoT solutions, requiring significant investment for large deployments.	Best suited for large enterprises needing highly customized industrial IoT solutions, potentially involving significant initial investment. Not typically designed for small or hobbyist projects.	3

**Table 7:** Comparison of developer tools and SDK support across IoT Cloud Platforms.

Platform	References	Key SDKs and Developer Resources	Score
Arduino IoT Cloud	[187,188,189,190,191,192,193]	Web-based Cloud Editor, Arduino CLI, auto-generated device sketches, REST APIs, Arduino/C++/JavaScript/Python SDKs.	8
AWS IoT Core	[194]	Comprehensive SDKs in Embedded C, C++, Python, JavaScript, Java. Mobile SDKs for Android and iOS. Rich CLI and extensive documentation.	9
Blynk	[195]	Blynk.Edgents for easy device connection. SDKs for mobile and embedded applications (portable C++ libraries for Arduino, ESP32, etc.).	7
Bosch IoT Suite	[196]	SDKs include Java and REST-like HTTP API. Supports open-source Eclipse IoT projects (Hono, Ditto, Vorto, hawkBit).	7
IoT on Google Cloud Platform	[197]	Previously supported through Google Cloud SDK and client libraries; largely deprecated as of August 2023. Reliance on generic Google Cloud libraries.	3
Microsoft Azure IoT Hub	[198]	Broad SDKs in C, .NET, Node.js, Java, and Python. Embedded SDKs for Eclipse ThreadX, FreeRTOS, and Bare Metal. Comprehensive Azure CLI.	9
Oracle IoT Cloud Service	[199]	SDKs in Java, JavaScript, C Posix, and REST API. Mobile SDKs for Android and iOS. Integrated developer tools.	7
Particle IoT Platform	[200]	Particle API JS (JavaScript SDK), hardware device SDKs, API libraries in Node.js and Python. Mobile SDKs for Android and iOS.	8
Siemens Insights Hub	[167,201]	SDKs include Java, Python, Node.js. Fleet Manager Plugin SDK. Offers Open Edge Device Kit and extensive APIs.	8
ThingsBoard	[202,203,204,205,206]	MQTT, CoAP, HTTP, LwM2M, SNMP APIs. REST APIs with Java, Python, Dart clients. Python Client SDK, Arduino SDK. Mobile application development (Flutter).	9
ThingSpeak	[64,207,208]	REST and MQTT APIs. MATLAB analytics integration. Arduino, ESP8266, ESP32, Raspberry Pi libraries. Web-based data visualization and analysis.	7
ThingWorx	[209]	ThingWorx Edge Java, .NET, and C SDKs. Comprehensive REST APIs and visual development tools.	8

**Table 8:** Comparison of compliance and standards across IoT Cloud Platforms.

Platform	References	Key Certifications and Standards Supported	Score
Arduino IoT Cloud	[210]	ISO/IEC 27001:2013	7
AWS IoT Core	[211]	ISO 27001. 27017. 27018. 27701. 22301. 20000-1. 9001; CSA STAR, CCM v4.0, SOC, PCI DSS, ISMAP, FINMA, FedRAMP, HIPAA, etc.	9
Blynk	[74]	SOC2 (in process); general data protection policies.	4
Bosch IoT Suite	[76]	ISO 27001, ISO 9001:2015, ISO/IEC 20000-1:2018, and TISAX.	7
IoT on Google Cloud Platform	[212]	ISO 27001. 27017. 27018. 27701. 22301. 20000-1. 9001; CSA STAR, SOC1/2/3, PCI DSS, ISMAP, FINMA, FIPS 140-2, FedRAMP, HIPAA, NIST, etc.	9
Microsoft Azure IoT Hub	[213]	ISO 27001. 27017. 27018. 27701. 22301. 20000-1. 9001; CSA STAR, SOC1/2/3, PCI DSS, ISMAP, FINMA, FIPS 140-2, FedRAMP, HIPAA, NIST, etc.	9
Oracle IoT Cloud Service	[214]	ISO 27001. 27017. 27018. 27701. 20000-1. 9001; CSA STAR, SOC1/2/3, PCI DSS, ISMAP, FedRAMP, NIST, etc.	9
Particle IoT Platform	[215]	ISO 27001. 27017. 27018, SOC 2.	7
Siemens Insights Hub	[216]	ISO 27001, IEC 62443-4-1 (Industrial Cybersecurity Standard).	7
ThingsBoard	[217,218]	Robust security measures including role-based access control, various device authentication options (Access Tokens, Basic MQTT Credentials, X.509 Certificates), and support for MQTT over SSL/TLS. GDPR compliant.	8
ThingSpeak	[219,220]	Uses secure communication (SSL/TLS) with certificates (DigiCert Global Root CA). Offers API keys for channel read/write access.	6
ThingWorx	[221]	ISO 27001:2013, SOC 2.	6

**Table 9:** Comparison of OTA update capabilities across IoT Cloud Platforms.

Platform	References	Key Features	Supported Device Platforms/Ecosystems	Score
Arduino IoT Cloud	[223,224,225,226,227]	The Update process is managed via Web interface or the Arduino CLI using Arduino Sketches. Enterprise users gain access to fleet-wide firmware rollout features and secure OTA updates for devices like the high-performance board Portenta X8, which runs Linux OS (Yocto). Very flexible OTA platform.	ESP32, ESP8266, Arduino MKR, Nano, Porenta, Nicla, etc.	9
AWS IoT Core	[228]	AWS IoT Device Management supports secure, scalable OTA updates using AWS IoT Jobs. Integration with FreeRTOS OTA agent for secure firmware updates.	FreeRTOS-based devices; general support for devices integrated with AWS IoT Device SDK for Embedded C.	9
Blynk	[229]	Blynk.Air provides remote firmware updates with device-side checks and basic rollback support; primarily Wi-Fi connectivity.	ESP32, ESP8266, Seeed Wio Terminal, Arduino MKR1010, Arduino Nano 33 IoT, Texas Instruments CC3220, etc.	7
Bosch IoT Suite	[230]	Bosch IoT Rollouts offers comprehensive SOTA (Software updates over the air) management including secure delivery, campaign management, and rollback features.	Wide range of devices via dedicated agents; strong focus on industrial/Bosch-specific devices.	8
IoT on Google Cloud Platform	[58]	Google Cloud IoT Core is officially deprecated. While data might be sent to other Google Cloud services, no dedicated OTA update service as part of the IoT Core offering.	Currentlly not availiable. Deprecated Service.	3
Microsoft Azure IoT Hub	[231]	Device Update for IoT Hub enables deployment of OTA updates for IoT devices at scale, with robust management, monitoring, and rollback capabilities.	Azure RTOS, Linux, and Eclipse ThreadX; extensible to other OSes via custom agents.	9
Oracle IoT Cloud Service	[232,233]	Includes OTA management within device administration for secure firmware updates; capabilities for deployment, monitoring, and basic version control. Limited documented rollback features.	Devices connected via Oracle IoT Device SDKs (e.g., Java, C Posix).	7
Particle IoT Platform	[234]	Provides extensive OTA update capabilities tightly integrated with Particle Device OS, ensuring secure and reliable firmware updates for their hardware ecosystem.	Particle Device OS and Particle hardware (SoMs, boards).	8
Siemens Insights Hub	[167]	Features OTA update tools specifically designed for Siemens’ industrial devices, enabling secure firmware and software updates within industrial control systems.	Siemens Industrial Devices and MindConnect gateways.	7
ThingsBoard	[235]	Supports remote firmware and software OTA updates, allowing package management, assignment to device profiles or individual devices, tracking update status, and basic queuing for large-scale rollouts.	Devices capable of MQTT, HTTP, CoAP, or LwM2M communication, with specific guides for ESP32 devices.	8
ThingSpeak	[207]	Primarily an IoT analytics platform, ThingSpeak’s OTA capabilities are typically managed via external device-side code (e.g., Arduino sketches for ESP8266/ESP32) that interacts with ThingSpeak to fetch firmware URLs.	ESP8266, ESP32, Arduino boards, and Particle devices through their respective libraries and custom implementations.	5
ThingWorx	[236]	Includes robust OTA support through ThingWorx Device Management, offering secure update deployment, monitoring of update status, and limited rollback functionality.	Devices connected via ThingWorx Edge SDKs (Java, .NET, C) or supported industrial protocols.	9

**Table 10:** Comprehensive feature-driven scores of IoT Cloud Platforms (scores: 1–10).

Platforms	Security	Scalability and Performance	Interoperability	Data Analytics and AI/ML Integration	Edge Computing Support	Pricing Models and Cost-Effectiveness	Developer Tools and SDK Support	Compliance and Standards	OTA Update Capabilities
**Arduino IoT Cloud**	8	8	9	7	7	6	8	7	9
**AWS IoT Core**	10	10	8	10	9	9	9	9	9
**Blynk**	5	6	7	4	2	6	7	4	7
**Bosch IoT SuiteBosch IoT Suite**	8	8	7	7	7	5	7	7	8
**IoT on Google Cloud Platform**	10	9	9	10	9	9	3	9	3
**Microsoft Azure IoT Hub**	10	9	8	10	9	9	9	9	9
**Oracle IoT Cloud Service**	8	8	7	7	7	4	7	9	7
**Particle IoT Platform**	7	6	5	5	7	7	8	7	8
**Siemens Insights Hub**	6	8	7	8	7	3	8	7	7
**ThingsBoard**	8	9	8	8	8	9	9	8	8
**ThingSpeak**	6	5	6	7	4	7	7	6	5
**ThingWorx**	7	8	9	8	7	3	8	6	9

Note: The assigned score (1–10) for each feature and platform is based on our research at the time of publication.

## Data Availability

The source code used to support the findings of this study is available at https://github.com/TelSiP-RLab/IoT-Cloud-Platforms-Selector (accessed on 11 August 2025). The online web-based tool for evaluating IoT Cloud Platforms is available at https://telsip-rlab.github.io/IoT-Cloud-Platforms-Selector (accessed on 11 August 2025). In this study, no new data were generated.

## Abbreviations

The following abbreviations are used in this manuscript:

2FA	Two-Factor Authentication
AES	Advanced Encryption Standard
AI	Artificial Intelligence
AMQP	Advanced Message Queuing Protocol
API	Application Programming Interface
AR	Augmented Reality
AWS	Amazon Web Services
CBOR	Concise Binary Object Representation
CIA	Confidentiality, Integrity, and Availability
CLI	Command Line Interface
CoAP	Constrained Application Protocol
CSA	Cloud Security Alliance
CSV	Comma Separated Values
CVSS	Common Vulnerability Scoring System
DevNet	Developer Network
DTLS	Datagram Transport Layer Security
EMS	Edge MicroServer
ETSI	European Telecommunications Standards Institute
FedRAMP	Federal Risk and Authorization Management Program
FINMA	Swiss Financial Market Supervisory Authority
FIPS	Federal Information Processing Standard
FreeRTOS	Free Real-Time Operating System
GCP	Google Cloud Platform
GDPR	General Data Protection Regulation
HIPAA	Health Insurance Portability and Accountability Act
HTTP	Hypertext Transfer Protocol
HTTPS	HyperText Transfer Protocol Secure
IAM	Identity and Access Management
IDE	Integrated Development Environment
IIoT	Industrial Internet of Things
iOS	iPhone Operating System
IoT	Internet of Things
IP	Internet Protocol
ISO	International Organization for Standardization
Java EE	Java platform Enterprise Edition
JMS	Java Message Service
JS	JavaScript
JSON	JavaScript Object Notation
LDAP	Lightweight Directory Access Protocol
LTPA	Lightweight Third Party Authentication
M2M	Machine-to-Machine
MFA	Multi-Factor Authentication
ML	Machine Learning
MQTT	Message Queuing Telemetry Transport
OAuth	Open Authorization
ODBC	Open DataBase Connectivity
oneM2M	One Machine-to-Machine
OPC	Open Platform Communications
OT	Operational Technology
OTA	Over-The-Air
Paas	Platform-as-a-Service
PCI DSS	Payment Card Industry Data Security Standard
PoC	Proof-of-Concept
PTC	Parametric Technology Corporation
REST	REpresentational State Transfer
ROI	Return on Investment
SDK	Software Development Kit
SMEs	Small and Medium-sized Enterprises
SNMP	Simple Network Management Protocol
SoMs	System-on-Modules
SSL	Secure Sockets Layer
SSO	Single Sign-On
STAR	Security, Trust, Assurance, and Risk
STRIDE	Spoofing, Tampering, Repudiation, Information Disclosure, Denial of Service, and Elevation of Privilege
TCO	Total Cost of Ownership
TCP	Transmission Control Protocol
TLS	Transport Layer Security
TPM	Trusted Platform Module
UDP	User Datagram Protocol
UI	User Interface
URL	Uniform Resource Locator
W3C	World Wide Web Consortium
WSNs	Wireless Sensor Networks
XMPP	eXtensible Messaging and Presence Protocol

## References

[1] Kizza J.M. (2024). Internet of things (iot): Growth, challenges, and security. Guide to Computer Network Security.

[2] Botta A., De Donato W., Persico V., Pescapé A. (2016). Integration of cloud computing and internet of things: A survey. Future Gener. Comput. Syst..

[3] Lin J., Yu W., Zhang N., Yang X., Zhang H., Zhao W. (2017). A survey on internet of things: Architecture, enabling technologies, security and privacy, and applications. IEEE Internet Things J..

[4] Gubbi J., Buyya R., Marusic S., Palaniswami M. (2013). Internet of Things (IoT): A vision, architectural elements, and future directions. Future Gener. Comput. Syst..

[5] Ahmed S.F., Alam M.S.B., Hoque M., Lameesa A., Afrin S., Farah T., Kabir M., Shafiullah G., Muyeen S. (2023). Industrial Internet of Things enabled technologies, challenges, and future directions. Comput. Electr. Eng..

[6] Sikarwar R., Yadav P., Dubey A. (2020). A Survey on IOT enabled cloud platforms. Proceedings of the 2020 IEEE 9th International Conference on Communication Systems and Network Technologies (CSNT).

[7] Sharma B., Obaidat M.S. (2020). Comparative analysis of IoT based products, technology and integration of IoT with cloud computing. IET Netw..

[8] Hamdan S., Ayyash M., Almajali S. (2020). Edge-computing architectures for internet of things applications: A survey. Sensors.

[9] Haseeb-Ur-Rehman R.M.A., Liaqat M., Aman A.H.M., Ab Hamid S.H., Ali R.L., Shuja J., Khan M.K. (2021). Sensor cloud frameworks: State-of-the-art, taxonomy, and research issues. IEEE Sens. J..

[10] Kondratenko Y., Kondratenko G., Sidenko I. (2018). Multi-criteria decision making for selecting a rational IoT platform. Proceedings of the 2018 IEEE 9th International Conference on Dependable Systems, Services and Technologies (Dessert).

[11] Ballamudi S. (2025). Evaluating IoT Platforms: An Approach Using the COPRAS Method. J. Data Sci. Inf. Technol..

[12] Al-Fuqaha A., Guizani M., Mohammadi M., Aledhari M., Ayyash M. (2015). Internet of things: A survey on enabling technologies, protocols, and applications. IEEE Commun. Surv. Tutor..

[13] Muhammed A.S., Ucuz D. (2020). Comparison of the IoT platform vendors, microsoft Azure, Amazon web services, and Google cloud, from users’ perspectives. Proceedings of the 2020 8th International Symposium on Digital Forensics and Security (ISDFS).

[14] Minoli D., Sohraby K., Occhiogrosso B. (2017). IoT considerations, requirements, and architectures for smart buildings—Energy optimization and next-generation building management systems. IEEE Internet Things J..

[15] Monios N., Peladarinos N., Cheimaras V., Papageorgas P., Piromalis D.D. (2024). A thorough review and comparison of commercial and open-source IoT platforms for smart city applications. Electronics.

[16] Surianarayanan C., Chelliah P.R. (2023). Integration of the internet of things and cloud: Security challenges and solutions—A review. Int. J. Cloud Appl. Comput. (IJCAC).

[17] Perera C., Zaslavsky A., Christen P., Georgakopoulos D. (2013). Context aware computing for the internet of things: A survey. IEEE Commun. Surv. Tutor..

[18] Ullah M., Kakakhel S.R.U., Westerlund T., Wolff A., Carrillo D., Plosila J., Nardelli P.H. (2020). Iot protocol selection for smart grid applications: Merging qualitative and quantitative metrics. Proceedings of the 2020 43rd International Convention on Information, Communication and Electronic Technology (MIPRO).

[19] Guth J., Breitenbücher U., Falkenthal M., Fremantle P., Kopp O., Leymann F., Reinfurt L. (2018). A detailed analysis of IoT platform architectures: Concepts, similarities, and differences. Internet of Everything: Algorithms, Methodologies, Technologies and Perspectives.

[20] Ilieva G., Yankova T. (2022). IoT system selection as a fuzzy multi-criteria problem. Sensors.

[21] Mayer B., Lackner K.M. Selection of an IoT Platform: A Framework for a Two-Stage Multi-Criteria Decision Making Process. Proceedings of the 2022 8th International Conference on Computer Technology Applications.

[22] Ganguly P. (2016). Selecting the right IoT cloud platform. Proceedings of the 2016 International Conference on Internet of Things and Applications (IOTA).

[23] Astropekakis K., Drakakis E., Grammatikakis K., Goumopoulos C. (2022). A survey of IoT software platforms. Advances in Computing, Informatics, Networking and Cybersecurity: A Book Honoring Professor Mohammad S. Obaidat’s Significant Scientific Contributions.

[24] Ray P.P. (2016). A survey of IoT cloud platforms. Future Comput. Inform. J..

[25] Babun L., Denney K., Celik Z.B., McDaniel P., Uluagac A.S. (2021). A survey on IoT platforms: Communication, security, and privacy perspectives. Comput. Netw..

[26] Mahmoud R., Yousuf T., Aloul F., Zualkernan I. (2015). Internet of things (IoT) security: Current status, challenges and prospective measures. Proceedings of the 2015 10th international conference for internet technology and secured transactions (ICITST).

[27] Bertino E., Islam N. (2017). Botnets and internet of things security. Computer.

[28] Yu J.Y., Kim Y.G. (2019). Analysis of IoT platform security: A survey. Proceedings of the 2019 International Conference on Platform Technology and Service (PlatCon).

[29] Fortino G., Guerrieri A., Pace P., Savaglio C., Spezzano G. (2022). Iot platforms and security: An analysis of the leading industrial/commercial solutions. Sensors.

[30] Ali A.H. (2019). A survey on vertical and horizontal scaling platforms for big data analytics. Int. J. Integr. Eng..

[31] Noura M., Atiquzzaman M., Gaedke M. (2019). Interoperability in internet of things: Taxonomies and open challenges. Mob. Netw. Appl..

[32] Palau C.E., Fortino G., Montesinos M., Exarchakos G., Giménez P., Markarian G., Castay V., Fuart F., Pawłowski W., Mortara M. (2021). Interoperability of Heterogeneous IoT Platforms.

[33] Duan L., Da Xu L. (2024). Data analytics in industry 4.0: A survey. Inf. Syst. Front..

[34] Panduman Y.Y.F., Funabiki N., Fajrianti E.D., Fang S., Sukaridhoto S. (2024). A survey of AI techniques in IoT applications with use case investigations in the smart environmental monitoring and analytics in real-time IoT platform. Information.

[35] Kumar K., Kumar V., Seema. (2023). Integration of Artificial Intelligence and Machine Learning for Internet of Things. Proceedings of the 2023 International Conference on Sustainable Communication Networks and Application (ICSCNA).

[36] Uddin M.K.S. (2024). A Review of Utilizing Natural Language Processing and AI For Advanced Data Visualization in Real-Time Analytics. Glob. Mainstream J..

[37] Kong L., Tan J., Huang J., Chen G., Wang S., Jin X., Zeng P., Khan M., Das S.K. (2022). Edge-computing-driven internet of things: A survey. ACM Comput. Surv..

[38] Jassim M.M., Mosa M.M., Okbi Z.A.I., Abdullah S.B., Taha S.W., Migo P., Kondakova S. (2024). Cost-Effectiveness Analysis of IoT Deployment in 5G Networks. Proceedings of the 2024 36th Conference of Open Innovations Association (FRUCT).

[39] Ghafari F., Shourangiz E., Wang C. (2024). Cost effectiveness of the Industrial Internet of Things adoption in the US manufacturing SMEs. Intell. Sustain. Manuf..

[40] Larrucea X., Combelles A., Favaro J., Taneja K. (2017). Software engineering for the internet of things. IEEE Softw..

[41] Uddin G., Sabir F., Guéhéneuc Y.G., Alam O., Khomh F. (2021). An empirical study of IoT topics in IoT developer discussions on Stack Overflow. Empir. Softw. Eng..

[42] Lee E., Seo Y.D., Oh S.R., Kim Y.G. (2021). A Survey on Standards for Interoperability and Security in the Internet of Things. IEEE Commun. Surv. Tutor..

[43] Catuogno L., Galdi C. (2023). Secure Firmware Update: Challenges and Solutions. Cryptography.

[44] Bakhshi T., Ghita B., Kuzminykh I. (2024). A review of IoT firmware vulnerabilities and auditing techniques. Sensors.

[45] Koo M., Yang S.W. (2025). Likert-Type Scale. Encyclopedia.

[46] Bresciani S., Eppler M.J. (2008). Gartner’s magic quadrant and hype cycle. Inst. Mark. Commun. Manag. (IMCA) Univ. Della Svizz. Ital. Fac. Commun. Sci. Case.

[47] Pelino M., Hewitt A. (2016). The FORRESTER Wave™: IoT Software Platforms, Q4 2016. https://www.tca.pt/pdf/RES136087.pdf.

[48] Skjong R., Wentworth B.H. (2001). Expert judgment and risk perception. Proceedings of the ISOPE International Ocean and Polar Engineering Conference.

[49] Arduino IoT Cloud (2025). Arduino IoT Cloud. https://docs.arduino.cc/arduino-cloud/.

[50] Arduino (2025). Arduino. https://arduino.cc/.

[51] Espressif (2025). Espressif. https://www.espressif.com/en/products/socs/esp32/.

[52] Edge Impulse (2025). Edge Impulse. https://docs.edgeimpulse.com/docs/integrations/arduino-mltools.

[53] Arduino Cloud Enterprise (2025). Arduino Cloud Enterprise. https://aws.amazon.com/marketplace/pp/prodview-zoqw3a4xgw4o6.

[54] Arduino Cloud Enterprise (2025). Cisco IoT Control Center—Cloud Connect Solution Overview. https://www.cisco.com/c/en/us/solutions/collateral/internet-of-things/iot-control-center/iot-control-center-cloud-con-so.html.

[55] AWS IoT Core (2025). AWS IoT Core. https://aws.amazon.com/iot-core/.

[56] Blynk (2025). Blynk. https://blynk.io/.

[57] Bosch IoT Suite (2025). Bosch IoT Suite. https://bosch-iot-suite.com/.

[58] Google Cloud (2025). IoT Platform Product Architecture on Google Cloud. https://cloud.google.com/architecture/connected-devices/iot-platform-product-architecture.

[59] Azure IoT Hub (2025). Azure IoT Hub. https://azure.microsoft.com/en-us/products/iot-hub.

[60] Oracle IoT Cloud Service (2025). Oracle IoT Cloud Service. https://docs.oracle.com/en/cloud/paas/iot-cloud/.

[61] Particle IoT Platform (2025). Particle IoT Platform. https://www.particle.io/.

[62] Siemens MindSphere (2025). Siemens MindSphere. https://documentation.mindsphere.io/MindSphere/index.html.

[63] ThingsBoard (2025). ThingsBoard. https://thingsboard.io/.

[64] MathWorks (2025). MathWorks. https://thingspeak.mathworks.com/.

[65] MathWorks (2025). MatLab. https://www.mathworks.com/products/matlab.html.

[66] ThingWorx IIoT Platform (2025). ThingWorx IIoT Platform. https://www.ptc.com/en/products/thingworx.

[67] Szymoniak S., Kesar S. (2022). Key agreement and authentication protocols in the internet of things: A survey. Appl. Sci..

[68] Arduino IoT Cloud (2025). How IoT Device Provisioning to the Arduino IoT Cloud Works. https://blog.arduino.cc/2020/08/31/how-iot-device-provisioning-to-the-arduino-iot-cloud-works/.

[69] Arduino IoT Cloud (2025). Supported Arduino Cloud Devices. https://support.arduino.cc/hc/en-us/articles/360016077320-Supported-Arduino-Cloud-devices.

[70] Arduino IoT Cloud (2025). Arduino Cloud Services Are ISO 27001 Certified. https://docs.arduino.cc/arduino-cloud/business/iso27001/.

[71] Arduino IoT Cloud (2025). Arduino Cloud Security Considerations. https://docs.arduino.cc/arduino-cloud/business/security-considerations.

[72] Amazon Web Services IoT Core (2025). Transport Security in AWS IoT Core—AWS IoT Core. https://docs.aws.amazon.com/iot/latest/developerguide/transport-security.html.

[73] Amazon Web Services IoT Core (2025). AWS IoT Security. https://docs.aws.amazon.com/iot/latest/developerguide/iot-security.html.

[74] Blynk (2025). Blynk Documentation—Security. https://docs.blynk.io/en/blynk.cloud/security.

[75] Blynk (2025). Blynk Documentation—Authentication. https://docs.blynk.io/en/blynk.cloud/platform-https-api/authentication.

[76] Bosch IoT Insights (2025). Bosch IoT Insights—Introduction. https://bosch-iot-insights.com/static-contents/docu/html/Introduction.html.

[77] Google Cloud (2024). Standalone MQTT Broker Architecture on Google Cloud. https://cloud.google.com/architecture/connected-devices/mqtt-broker-architecture.

[78] Google Cloud (2025). Authentication Methods at Google. https://cloud.google.com/docs/authentication.

[79] Gremban K., Kennedy D., Brannian J., Malhotra R., Lian J., Guo M., Sherafat R., Shahan R. (2025). Transport Layer Security (TLS) Support in IoT Hub. https://learn.microsoft.com/en-us/azure/iot-hub/iot-hub-tls-support.

[80] Betts D., Lt T., Heinrich A., Gremban K., McSwain W. (2025). Security Practices for Azure IoT Device Manufacturers. https://learn.microsoft.com/en-us/azure/iot-dps/concepts-device-oem-security-practices.

[81] Oracle (2025). REST API for Oracle Internet of Things Cloud Service. https://docs.oracle.com/en/cloud/paas/iot-cloud/iotrq/Authentication.html.

[82] Sara A., Randa J. (2024). Data protection in IoT using CoAP based on enhanced DTLS. Proceedings of the 16th International Engineering and Computing Research Conference (Eureca).

[83] Particle IoT Platform (2025). Device Cloud Introduction. https://docs.particle.io/getting-started/cloud/introduction/.

[84] Particle IoT Platform (2025). Cloud API Reference. https://docs.particle.io/reference/cloud-apis/api/.

[85] Siemens MindSphere (2025). MindConnect MQTT Broker. https://documentation.mindsphere.io/MindSphere/concepts/concept-mindconnect-mqtt-broker.html.

[86] Siemens MindSphere (2025). Agent Management Service. https://documentation.mindsphere.io/MindSphere/apis/connectivity-agentmanagement/api-agentmanagement-overview.html.

[87] ThingsBoard (2025). ThingsBoard Security. https://thingsboard.io/docs/mqtt-broker/security/.

[88] ThingsBoard (2025). ThingsBoard Architecture. https://thingsboard.io/docs/reference/architecture/.

[89] ThingsBoard (2025). ThingsBoard—Two-Factor Authentication. https://thingsboard.io/docs/user-guide/two-factor-authentication/.

[90] MathWorks (2025). System Requirements. https://www.mathworks.com/products/thingspeak/system-requirements.html.

[91] MathWorks (2025). User Accounts and Channels: ThingSpeak API Keys. https://www.mathworks.com/help/thingspeak/users-and-channels.html.

[92] ThingWorx IIoT Platform (2025). ThingWorx Edge C SDK—Using SSL/TLS for Security. https://support.ptc.com/help/thingworx/edge_sdk_c/en/index.html#page/c_sdk/c_csdk_tls_implementation.html.

[93] ThingWorx IIoT Platform (2025). ThingWorx Platform—Security. https://support.ptc.com/help/thingworx/platform/r9.6/en/index.html#page/ThingWorx/Help/Composer/Security/Security.html.

[94] Luntovskyy A., Globa L. (2019). Performance, reliability and scalability for IoT. Proceedings of the 2019 International Conference on Information and Digital Technologies (IDT).

[95] Arduino Cloud (2025). Arduino and AWS Team up to Bridge Hardware and Cloud for Business. https://blog.arduino.cc/2023/08/31/arduino-and-aws-team-up-to-bridge-hardware-and-cloud-for-business/.

[96] Arduino Cloud (2025). Endless IoT Possibilities for Your Business. https://cloud.arduino.cc/cloud-for-business/.

[97] Amazon Web Services IoT Core (2025). AWS IoT Core Endpoints and Quotas. https://docs.aws.amazon.com/general/latest/gr/iot-core.html#message-broker-limits.

[98] Blynk (2025). Blynk Limits. https://docs.blynk.io/en/blynk.console/limits.

[99] Bosch IoT Devices (2025). Boch IoT Device Management—Restrictions Related to the Device Connectivity Layer. https://docs.bosch-iot-suite.com/device-management/Restrictions-related-to-the-device-connectivity-layer.html.

[100] ReadITQuik (2018). Google Announces New Cloud IoT Core Commands. https://readitquik.com/cloud-3/google-announces-new-cloud-iot-core-commands/.

[101] Gremban K., Kennedy D., Betts D., Brannian J., Lian J., Shahan R., Wick S., Huygen v.M., Jenks A., Guo M. (2025). IoT Hub Quotas and Throttling. https://learn.microsoft.com/en-us/azure/iot-hub/iot-hub-devguide-quotas-throttling.

[102] Oracle (2022). Oracle IoT Intelligent Applications for Energy and Water. https://www.oracle.com/a/ocom/docs/industries/iot-intelligent-applications-ds.pdf.

[103] Particle IoT Platform (2025). Billing—Data Operations. https://docs.particle.io/getting-started/billing/data-operations/.

[104] Siemens MindSphere (2025). Industrial IoT Gateway—Developer Documentation. https://documentation.mindsphere.io/MindSphere/concepts/concept-gateway-url-schemas.html#restrictions.

[105] ThingsBoard (2025). ThingsBoard Cloud Subscription Plans Definition. https://thingsboard.io/docs/paas/subscription/.

[106] ThingSpeak (2025). ThingSpeak™ Licensing FAQ. https://thingspeak.mathworks.com/pages/license_faq.

[107] ThingSpeak (2025). ThingSpeak™ Standard License. https://thingspeak.mathworks.com/prices/thingspeak_standard.

[108] ThingWorx IIoT Platform (2019). ThingWorx Platform 8.x—Sizing Guide. https://community.ptc.com/sejnu66972/attachments/sejnu66972/twxdevs/55157/1/ThingWorx_Platform_8_x_Sizing_Guide.pdf.

[109] Albouq S.S., Abi Sen A.A., Almashf N., Yamin M., Alshanqiti A., Bahbouh N.M. (2022). A survey of interoperability challenges and solutions for dealing with them in IoT environment. IEEE Access.

[110] Arduino Cloud (2025). An Intro to the Arduino Cloud. https://docs.arduino.cc/learn/starting-guide/arduino-iot-cloud/.

[111] Arduino Cloud (2025). Arduino IoT Cloud API. https://docs.arduino.cc/cloud-api/.

[112] Arduino Cloud (2025). Webhooks. https://docs.arduino.cc/arduino-cloud/features/webhooks/.

[113] Arduino Cloud (2025). Alexa. https://docs.arduino.cc/arduino-cloud/guides/alexa/.

[114] Arduino Cloud (2025). Arduino Cloud Amazon Alexa Integration. https://docs.arduino.cc/tutorials/projects/arduino-iot-cloud-amazon-alexa-integration/.

[115] Arduino Cloud (2025). Node-RED. https://docs.arduino.cc/arduino-cloud/guides/node-red/.

[116] Arduino Cloud (2025). Google Home. https://docs.arduino.cc/arduino-cloud/guides/google-home/.

[117] AWS IoT Core (2025). Device Communication Protocols. https://docs.aws.amazon.com/iot/latest/developerguide/protocols.html.

[118] Blynk (2023). Blynk Protocol. https://docs.blynk.io/en/blynk-library-firmware-api/blynk-protocol.

[119] Shymanskyy V. (2024). Blynk Expands Protocol Range: Introducing MQTT Support. https://blynk.io/blog/blynk-expands-protocol-range-introducing-mqtt-support.

[120] Bosch (2025). Bosch IoT Device Management. https://docs.bosch-iot-suite.com/asset-communication/Connections.html.

[121] Microsft Azure (2025). Microsoft Learn—Choose a Device Communication Protocol. https://learn.microsoft.com/en-us/azure/iot-hub/iot-hub-devguide-protocols.

[122] Oracle (2025). About the IoT Connectivity Protocols. https://docs.oracle.com/en/cloud/paas/iot-cloud/develop/iot-connectivity-protocols.html.

[123] Particle (2024). A 2024 Guide to IoT Protocols and Standards. https://www.particle.io/iot-guides-and-resources/iot-protocols-and-standards/.

[124] Siemens MindSphere (2023). MindConnect MQTT Introduction. https://documentation.mindsphere.io/MindSphere/connectivity/mindconnect-mqtt/mqtt-index.html.

[125] ThingsBoard (2025). Device Connectivity Protocols. https://thingsboard.io/docs/reference/protocols/.

[126] ThingsBoard (2025). MQTT Device API Reference. https://thingsboard.io/docs/reference/mqtt-api/.

[127] Viegas V., Dias Pereira J.M., Girão P., Postolache O. (2021). Study of latencies in ThingSpeak. Adv. Sci. Technol. Eng. Syst. J..

[128] ThingWorx (2024). ThingWorx Supported Protocols. https://www.ptc.com/en/support/article/CS248691.

[129] Al-Masri E., Kalyanam K.R., Batts J., Kim J., Singh S., Vo T., Yan C. (2020). Investigating messaging protocols for the Internet of Things (IoT). IEEE Access.

[130] Padyana U.K., Rai H.P., Ogeti P., Fadnavis N.S., Patil G.B. (2023). AI and Machine Learning in Cloud-Based Internet of Things (IoT) Solutions: A Comprehensive Review and Analysis. Integr. J. Res. Arts Humanit..

[131] Arduino IoT Cloud (2025). Dashboards and Widgets. https://docs.arduino.cc/arduino-cloud/cloud-interface/dashboard-widgets/.

[132] Arduino IoT Cloud (2025). Download Historical Data. https://docs.arduino.cc/arduino-cloud/features/iot-cloud-historical-data/.

[133] Arduino IoT Cloud (2025). Machine Learning Tools. https://cloud.arduino.cc/machine-learning-tools/.

[134] Arduino IoT Cloud (2025). Get Started with Machine Learning on Arduino. https://docs.arduino.cc/tutorials/nano-33-ble-sense/get-started-with-machine-learning/.

[135] Google (2025). LiteRT for Microcontrollers. https://ai.google.dev/edge/litert/microcontrollers/overview.

[136] AWS (2022). Creating a Machine Learning-Powered REST API with Amazon API Gateway Mapping Templates and Amazon SageMaker. https://aws.amazon.com/blogs/machine-learning/creating-a-machine-learning-powered-rest-api-with-amazon-api-gateway-mapping-templates-and-amazon-sagemaker/.

[137] Microsoft (2021). Arduino Library for Azure IoT. https://techcommunity.microsoft.com/blog/iotblog/arduino-library-for-azure-iot/3034455.

[138] Blynk (2025). Blynk Enterprise—White Label IoT Solution. https://blynk.io/iot-platform-for-business.

[139] Blynk (2025). Strategic Imperatives for B2B IoT Platforms in 2025: Embracing AI, Security and Developer Ecosystems. https://blynk.io/blog/strategic-imperatives-for-b2b-iot-platforms-2025.

[140] Bosch IoT Suite (2025). Bosch IoT Insights: Easily Manage Your IoT Data in the Cloud. https://bosch-iot-suite.com/service/insights/.

[141] Bosch IoT Suite (2025). Predictive Maintenance 4.0. https://bosch-iot-suite.com/predictive-maintenance-4-0/.

[142] Google Cloud Platform (2025). AI and Machine Learning Solutions. https://cloud.google.com/solutions/ai.

[143] Google Cloud Platform (2025). Dataflow Documentation. https://cloud.google.com/dataflow/docs.

[144] Google Cloud Platform (2025). BigQuery: From Data Warehouse to Autonomous Data and AI Platform. https://cloud.google.com/bigquery.

[145] Particle (2025). Transform Your IoT Project with Edge ML. https://www.particle.io/edge-ml/.

[146] Particle (2025). Machine Learning. https://docs.particle.io/getting-started/machine-learning/machine-learning/.

[147] Siemens MindSphere (2025). Data Integration and Management. https://documentation.mindsphere.io/MindSphere/data-integration-and-management/overview.html.

[148] Siemens MindSphere (2025). Artificial Intelligence in IoT. https://resources.sw.siemens.com/en-US/article-insight-hubs-iot-includes-ai-for-everyone/.

[149] ThingsBoard (2025). Large Language Model (LLM) Configuration. https://thingsboard.io/docs/trendz/custom-ai-model-configuration/.

[150] ThingsBoard (2025). Smart Farming—IoT Agriculture Solutions. https://thingsboard.io/use-cases/smart-farming/.

[151] ThingSpeak (2025). ThingSpeak for IoT. https://thingspeak.mathworks.com/pages/commercial_learn_more.

[152] ThingWorx (2025). IoT Analytics: Turn Data into Valuable Insights. https://www.ptc.com/en/products/thingworx/iot-analytics.

[153] Quadrant Knowledge Solutions (2018). PTC ThingWorx is Positioned as 2018 Technology Leader in IoT Platform Market by Quadrant Knowledge Solutions. https://quadrant-solutions.com/wp-content/uploads/2018/10/Knowledge-Brief_PTC-ThingWorx_IoT-Platforms-Market_2018-Technology-Leader_Quadrant-Knowledge-Solutions.pdf.

[154] Cao K., Liu Y., Meng G., Sun Q. (2020). An Overview on Edge Computing Research. IEEE Access.

[155] Arduino Cloud (2025). Arduino PRO: Edge IoT Technology. https://www.arduino.cc/pro/.

[156] Arduino Cloud (2025). Arduino’s Series of High-Performance Industry-Rated Boards. https://www.arduino.cc/pro/hardware-product-family-portenta-family/.

[157] Arduino Cloud (2025). Arduino’s Tiniest Industrial-Oriented Board. https://www.arduino.cc/pro/hardware-nicla-family/.

[158] Arduino Cloud (2025). The Ideal Solution for Emerging Battery Powered IoT Edge Applications. https://www.arduino.cc/pro/hardware-product-family-mkr-family/.

[159] AWS IoT Core (2025). AWS IoT for the Edge. https://aws.amazon.com/iot/solutions/iot-edge/.

[160] Blynk (2025). Blynk Documentation. https://docs.blynk.io/.

[161] Blynk (2025). Blynk Server (GitHub Repository)—Local Server Documentation. https://github.com/Peterkn2001/blynk-server.

[162] Blynk (2025). Blynk.Edgent Overview. https://docs.blynk.io/en/blynk.edgent/overview.

[163] Bosch IoT Suite (2025). IoT Edge: Empower the Intelligent Edge. https://bosch-iot-suite.com/iot-edge/.

[164] Google Cloud (2018). Bringing Intelligence to the Edge with Cloud IoT. https://cloud.google.com/blog/products/gcp/bringing-intelligence-edge-cloud-iot.

[165] Microsoft Azure (2025). Azure IoT Edge. https://azure.microsoft.com/en-us/products/iot-edge.

[166] Oracle (2025). Overview of Oracle Roving Edge Infrastructure. https://docs.oracle.com/en-us/iaas/Content/Rover/overview.htm.

[167] Siemens MindSphere (2025). Overview of MindConnect IoT2050. https://documentation.mindsphere.io/MindSphere/apps/mindconnect-iot2050/overview-of-mindconnect-iot2050.html.

[168] Siemens MindSphere (2025). Industrial Edge—Maximize Your Competitive Edge. https://www.siemens.com/global/en/products/automation/topic-areas/industrial-edge.html.

[169] ThingsBoard (2025). ThingsBoard Edge: Edge Computing. https://thingsboard.io/products/thingsboard-edge/.

[170] ThingsBoard (2025). ThingsBoard Edge: FAQ. https://thingsboard.io/docs/edge/faq/.

[171] ThingSpeak (2025). ThingSpeak|Data Collection in the Cloud with Advanced Data Analysis Using MATLAB. https://www.mathworks.com/help/thingspeak/.

[172] ThingWorx (2025). ThingWorx Edge MicroServer. https://support.ptc.com/help/thingworx/edge_microserver/en/index.html#page/c_sdk/c_ems_wsems_section_introduction.html.

[173] Arduino Cloud (2025). Arduino Cloud Plans. https://cloud.arduino.cc/plans/.

[174] AWS IoT Core (2025). AWS IoT Core Pricing. https://docs.aws.amazon.com/iot/latest/developerguide/iot-price.html.

[175] Blynk (2025). Blynk Pricing. https://blynk.io/pricing.

[176] Jordan Simeonov (2025). Bosch IoT Suite User Journey: Pricing. https://blog.bosch-digital.com/fourth-destination-pricing/.

[177] Bosch IoT Suite (2025). Pricing Calculator. https://docs.bosch-iot-suite.com/device-management/Pricing-calculator.html.

[178] Google Cloud (2025). Google Cloud Pricing. https://cloud.google.com/pricing.

[179] Microsoft Azure (2025). Azure IoT Hub Pricing. https://azure.microsoft.com/en-us/pricing/details/iot-hub/.

[180] Particle (2025). Particle—Pricing. https://www.particle.io/pricing/.

[181] Siemens MindSphere (2025). Industrial IoT. https://www.dex.siemens.com/?selected=industrial-iot.

[182] ThingsBoard (2025). Deploy ThingsBoard PE Maker. https://thingsboard.io/products/thingsboard-pe/install/aws/.

[183] ThingsBoard (2025). ThingsBoard Professional Edition. https://thingsboard.io/products/thingsboard-pe/.

[184] ThingSpeak (2025). License Options. https://thingspeak.mathworks.com/prices.

[185] ThingSpeak (2025). Home License. https://thingspeak.mathworks.com/prices/thingspeak_home.

[186] ThingWorx (2025). PTC Subscription. https://www.ptc.com/en/try-and-buy.

[187] Arduino Cloud (2025). Cloud Editor. https://docs.arduino.cc/arduino-cloud/guides/editor/.

[188] Arduino Cloud (2025). Arduino Cloud CLI. https://docs.arduino.cc/arduino-cloud/arduino-cloud-cli/getting-started/.

[189] Arduino Cloud (2025). Sketches. https://docs.arduino.cc/arduino-cloud/cloud-interface/sketches/.

[190] Arduino Cloud (2025). REST API and SDK. https://docs.arduino.cc/arduino-cloud/api/arduino-iot-api/.

[191] Arduino Cloud (2025). Arduino/C++ Library. https://docs.arduino.cc/arduino-cloud/api/c-library/.

[192] Arduino Cloud (2025). JavaScript/Node.js Library. https://docs.arduino.cc/arduino-cloud/api/javascript/.

[193] Arduino Cloud (2025). Python Client. https://docs.arduino.cc/arduino-cloud/api/python/.

[194] AWS IoT Core (2025). AWS IoT Device SDKs, Mobile SDKs, and AWS IoT Device Client. https://docs.aws.amazon.com/iot/latest/developerguide/iot-sdks.html.

[195] Blynk (2025). Blynk—Developers. https://blynk.io/developers.

[196] Bosch (2025). User Interfaces and Developer Tools. https://docs.bosch-iot-suite.com/device-management/User-interfaces-and-developer-tools.html.

[197] Google Cloud (2025). Google Cloud SDK. https://cloud.google.com/sdk/?hl=en.

[198] Microsoft Azure (2025). Azure IoT Hub SDKs. https://learn.microsoft.com/en-us/azure/iot-hub/iot-hub-devguide-sdks.

[199] Oracle (2025). Oracle IoT Cloud Service Client Software Library Downloads. https://www.oracle.com/downloads/cloud/iot-client-libraries-downloads.html.

[200] Particle (2025). Particle Docs—Reference. https://docs.particle.io/reference/reference/.

[201] Siemens MindSphere (2025). Siemens MindSphere Resources. https://documentation.mindsphere.io/resources/private-cloud/dev-docs/en-US/resources/index.html.

[202] ThingsBoard (2025). ThingsBoard API Reference. https://thingsboard.io/docs/api/.

[203] ThingsBoard (2025). Python Client SDK|ThingsBoard Community Edition. https://thingsboard.io/docs/reference/python-client-sdk/.

[204] ThingsBoard (2025). Java REST Client|ThingsBoard Community Edition. https://thingsboard.io/docs/reference/rest-client/.

[205] ThingsBoard (2025). Dart API Client|ThingsBoard Community Edition. https://thingsboard.io/docs/reference/dart-client/.

[206] ThingsBoard (2025). Thingsboard/Thingsboard-Arduino-Sdk: Arduino Client Library for ThingsBoard IoT Platform. https://github.com/thingsboard/thingsboard-arduino-sdk.

[207] MathWorks (2025). ThingSpeak Communication Library for Arduino, ESP8266 and ESP32—GitHub. https://github.com/mathworks/thingspeak-arduino/.

[208] MathWorks (2025). ThingSpeak API|Grafana Plugins Documentation. https://grafana.com/docs/plugins/yesoreyeram-infinity-datasource/latest/examples/thingspeak/.

[209] ThingWorx (2022). ThingWorx Edge SDKs. https://community.ptc.com/t5/IoT-Tips/ThingWorx-Edge-SDKs/ta-p/838628.

[210] Arduino Cloud (2023). Arduino Cloud Is ISO 27001 Certified. https://blog.arduino.cc/2023/08/02/arduino-cloud-is-iso-27001-certified/.

[211] AWS IoT Core (2025). AWS Services in Scope by Compliance Program. https://aws.amazon.com/compliance/services-in-scope/.

[212] Google Cloud (2025). Compliance Resource Center. https://cloud.google.com/compliance?hl=en.

[213] Microsoft Azure (2025). Center for Internet Security (CIS) Benchmarks. https://learn.microsoft.com/en-us/compliance/regulatory/offering-cis-benchmark.

[214] Oracle Cloud (2025). Oracle Cloud Compliance. https://www.oracle.com/de/corporate/cloud-compliance/.

[215] Particle (2025). IoT Security Done Right. https://www.particle.io/solutions/iot-security/.

[216] Siemen MindSpheres (2018). MindSphere Security Model. https://assets.new.siemens.com/siemens/assets/api/uuid:6b876b5e-5594-4da4-90e0-e9e0c6f1f1e1/version:1557483304/siemens-plm-mindsphere-security-model-wp-75966-a7.pdf.

[217] ThingsBoard (2025). Security Settings|ThingsBoard Professional Edition. https://thingsboard.io/docs/pe/user-guide/ui/security-settings/.

[218] ThingsBoard (2025). ThingsBoard Cloud Privacy Policy. https://thingsboard.io/products/paas/privacy-policy/.

[219] MathWorks (2025). Secure ThingSpeak Communications and Security Certificate. https://www.mathworks.com/matlabcentral/discussions/thingspeak/755925-secure-thingspeak-communications-and-security-certificate.

[220] MathWorks (2025). Channels—ThingSpeak. https://thingspeak.mathworks.com/channels/public?tag=MathWorks.

[221] ThingWorx (2025). Security Policies and Processes. https://support.ptc.com/help/windchill_engagement_guide/r12.1.2.0/en/index.html#page/windchill_engagement_guide/secure.html.

[222] Jimmy F. (2024). Cyber security Vulnerabilities and Remediation Through Cloud Security Tools. J. Artif. Intell. Gen. Sci. (JAIGS).

[223] Arduino Cloud (2025). No Cables Any More. https://cloud.arduino.cc/features-ota-updates/.

[224] Arduino Cloud (2025). OTA—Compatible Hardware. https://docs.arduino.cc/arduino-cloud/features/ota-getting-started/#compatible-hardware.

[225] Arduino Cloud (2025). Arduino Sketches. https://docs.arduino.cc/learn/programming/sketches/.

[226] Arduino Cloud (2025). Portenta X8. https://docs.arduino.cc/hardware/portenta-x8/.

[227] Arduino Cloud (2025). Getting Started with Arduino Cloud for Business. https://docs.arduino.cc/arduino-cloud/business/arduino-cloud-for-business/.

[228] AWS IoT Core (2025). Comparing AWS IoT Jobs and AWS IoT Over the Air (OTA) Updates. https://repost.aws/articles/ARDHNhV0bnRGau0kmdhTSZZA/comparing-aws-iot-jobs-and-aws-iot-over-the-air-ota-updates.

[229] Blynk (2023). OTA: Firmware Over-The-Air Updates. https://docs.blynk.io/en/blynk.edgent/updating-devices-firmwares-ota.

[230] Bosch (2025). Over-the-Air Updates: Key Considerations to Ensure a Secure Update Process. https://bosch-iot-suite.com/knowledge-center/expert-insights/ota-security/.

[231] Microsoft Azure (2024). What Is Device Update for IoT Hub?. https://learn.microsoft.com/en-us/azure/iot-hub-device-update/understand-device-update.

[232] Oracle (2025). Oracle Internet of Things. https://www.oracle.com/internet-of-things/.

[233] Oracle (2025). Developing Applications with Oracle Internet of Things Cloud Service. https://docs.oracle.com/en/cloud/paas/iot-cloud/iotgs/index.html.

[234] Particle (2025). OTA Firmware Updates. https://docs.particle.io/getting-started/cloud/ota-updates/.

[235] ThingsBoard (2025). ThingsBoard Over-the-Air (OTA) Updates. https://thingsboard.io/docs/user-guide/ota-updates/.

[236] PTC (2025). IoT Device Management. https://www.ptc.com/en/products/thingworx/iot-device-management.

